# Enhanced IL-2 in early life limits the development of T_FH_ and protective antiviral immunity

**DOI:** 10.1084/jem.20201555

**Published:** 2021-10-19

**Authors:** Chloe J. Pyle, Lucia Labeur-Iurman, Helen T. Groves, Franz Puttur, Clare M. Lloyd, John S. Tregoning, James A. Harker

**Affiliations:** 1 National Heart and Lung Institute, Imperial College London, South Kensington, London, UK; 2 Department of Infectious Disease, Imperial College London, St. Mary’s Campus, London, UK; 3 Asthma UK Centre in Allergic Mechanisms for Asthma, London, UK

## Abstract

T follicular helper cell (T_FH_)–dependent antibody responses are critical for long-term immunity. Antibody responses are diminished in early life, limiting long-term protective immunity and allowing prolonged or recurrent infection, which may be important for viral lung infections that are highly prevalent in infancy. In a murine model using respiratory syncytial virus (RSV), we show that T_FH_ and the high-affinity antibody production they promote are vital for preventing disease on RSV reinfection. Following a secondary RSV infection, T_FH_-deficient mice had significantly exacerbated disease characterized by delayed viral clearance, increased weight loss, and immunopathology. T_FH_ generation in early life was compromised by heightened IL-2 and STAT5 signaling in differentiating naive T cells. Neutralization of IL-2 during early-life RSV infection resulted in a T_FH_-dependent increase in antibody-mediated immunity and was sufficient to limit disease severity upon reinfection. These data demonstrate the importance of T_FH_ in protection against recurrent RSV infection and highlight a mechanism by which this is suppressed in early life.

## Teaser

Antibodies are central to long-term protection after infection or vaccination but often compromised in infancy. This study identifies a mechanism by which this compromise occurs and shows how it can be targeted to boost antibodies in early life and provide protection from reinfection.

## Introduction

Antibody-mediated immunity is central to long-term protection following natural infection or vaccination. The generation of high-affinity isotype-switched antibody predominantly takes place in germinal centers (GCs), anatomically distinct areas within the B cell zones of secondary lymphoid tissues. Within these GCs, B cells undergo somatic hypermutation and affinity maturation. A specialized subset of CD4 T cells, T follicular helper cells (T_FH_) control the proliferation, maturation, and survival of GC B cells (reviewed in Vinuesa et al., 2016). T_FH_ cells migrate into B cell follicles via a CXCL13–CXCR5 interaction, where they promote B cell proliferation and differentiation through costimulatory CD40L–CD40 signaling and the secretion of soluble mediators, most notably IL-21 and IL-4.

Expression of the transcriptional regulator Bcl6 is necessary and sufficient for T_FH_ generation (Johnston et al., 2009; Nurieva et al., 2009); deleting Bcl6 in T cells completely ablates T_FH_ formation (Hollister et al., 2013). In adult mice, the initial differentiation of T_FH_ requires multiple signals, including costimulation via CD28 and ICOS (Choi et al., 2011; Weber et al., 2015) and cytokine-induced STAT3 signaling (Ray et al., 2014). Conversely, STAT5 signaling can inhibit this process (Johnston et al., 2012; Nurieva et al., 2012). Sustained signaling through ICOS, CD28, and STAT3 is also involved in the maintenance and function of T_FH_ cells within GCs (Linterman et al., 2014; McIlwain et al., 2015; Weber et al., 2015; Wu et al., 2015).

T_FH_ cells are required for optimal antibody responses in a range of contexts, including murine models of vaccination, allergic inflammation, and acute and chronic viral infection (Dolence et al., 2018; Greczmiel et al., 2017; Harker et al., 2011; Hollister et al., 2013). Modulation of T_FH_ responses, for instance via ICOS–ICOS-L blockade or removal of IL-6 family cytokines, which signal through STAT3, can reduce antibody responses and alter isotype balance (Harker et al., 2013; Harker et al., 2015; Uwadiae et al., 2019). In humans, the expansion of functional circulating CXCR5^+^ PD1^+^ CD4^+^ T cells (surrogates of lymphoid T_FH_) correlates with the amplitude of antibody responses after influenza vaccination (He et al., 2013) and the emergence of broadly neutralizing antibodies in HIV-infected individuals (Locci et al., 2013). In contrast, individuals with loss-of-function mutations in key genes such as *ICOS* or *STAT3* have reduced humoral immunity and defects in the differentiation and function of their T_FH_ (Grimbacher et al., 2003; Ma et al., 2012).

Early life is often associated with a failure to respond robustly to vaccination or generate long-term protective immunity post-infection (p.i.). This is particularly true in the case of respiratory syncytial virus (RSV) infection, the most common cause of infant bronchiolitis in high-income countries and a virus for which there is no licensed vaccine (reviewed in Openshaw et al., 2017). Infants can be infected multiple times with the same serotype of RSV in their first 3 yr of life, and even in adults, protective levels of RSV-specific antibody responses are short-lived, and reinfection is common (Habibi et al., 2015). In mouse models, RSV-specific antibody responses are also limited after neonatal RSV infection compared with adults, characterized by reduced IgG (Tregoning et al., 2013). The underlying mechanism of this defect and whether the reduced humoral immune response contributes to disease on reinfection is currently unknown.

In the present study, the mechanisms underlying defects in early-life antibody-mediated immunity were explored. While RSV-infected adult mice generated robust antibody responses, those infected in early life failed to generate RSV-specific antibody responses. This failure to produce antibody was associated with limited differentiation of T_FH_ and GC B cells in the lung draining LNs (dLNs) and enhanced susceptibility to secondary infection. T_FH_-deficient mice highlighted the importance of these cells in the development of protective immunity during adult RSV infection. We also demonstrate that IL-2–dependent suppression of T_FH_ responses in early life is partially responsible for limiting antibody production, and reversing this process enhances protective immunity.

## Results

### RSV infection fails to generate T_FH_, GC B cells, and antibody in early life

**Figure S1.**
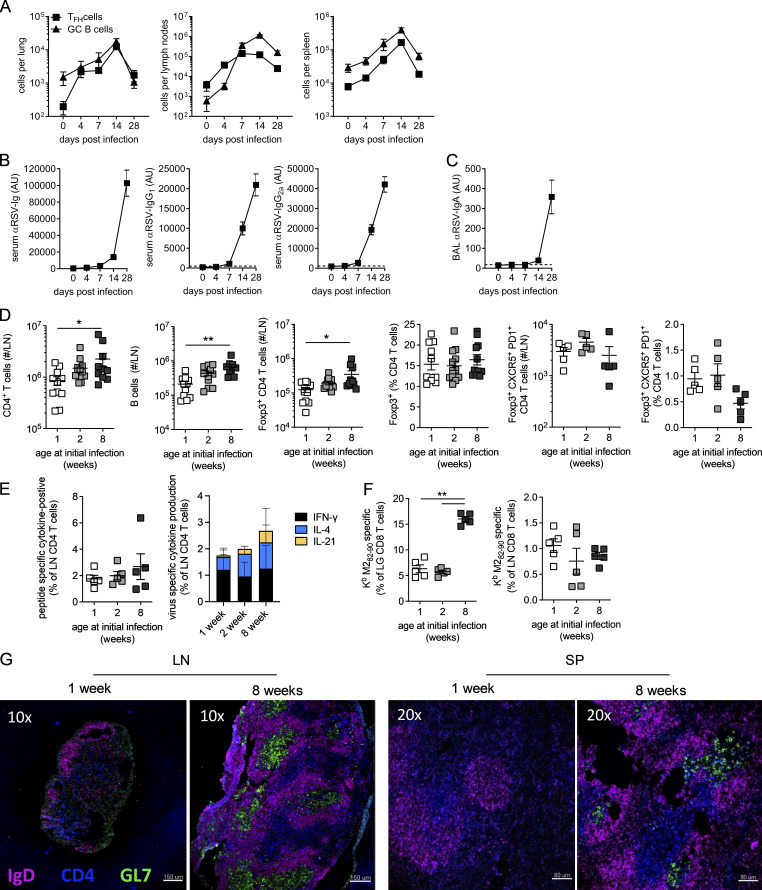
**Age-related reduction in adaptive immune responses to RSV infection.** 8-wk-old BALB/c mice were infected 8 × 10^5^ ffu RSV A2 i.n. **(A)** T_FH_ (CD19^−^CD8^−^Foxp3^−^CXCR5^+^PD1^+^CD49d^+^CD11a^+^CD4^+^) and GC B cell (IgD^−^IgM^−^CD38^−^GL7^+^CD19^+^) numbers were determined in the lungs, mediastinal LNs, and spleen p.i. **(B and C)** RSV-specific Ig, IgG_1_, and IgG_2a_ in the serum (B) and IgA in the airways (C). **(D–G)** 1-, 2-, and 8-wk-old BALB/c mice were infected 2.5 × 10^5^ ffu RSV A2 i.n. and sacrificed at day 14 p.i. **(D)** The total number of CD4 T cells, B cells, and Foxp3^+^ CD4 T cells was enumerated. **(E)** Cytokine production by LN CD4 T cells after RSV peptide stimulation. **(F)** Virus-specific CD8 T cells in the lungs and LNs. **(G)** LN and spleen (SP) from 1- and 8-wk-old mice were imaged by immunofluorescence microscopy for IgD, CD4, and GL7. Data are from *n* = 5–10 mice per time point and representative of two independent experiments, except in G, where a representative image from *n* = 6 mice per group across two independent repeats is shown. *, P < 0.05; and **, P < 0.01.

Since GC reactions are essential for a high-quality antibody response, we tested the role of age on the generation of GC responses to RSV. An initial characterization of the humoral immune response to RSV in 8-wk-old adult mice showed that T_FH_ and GC B cell frequencies peaked at day 14 p.i., primarily in the lung dLNs (Fig. S1 A). Circulating and airway virus-specific antibodies are detectable by day 7 p.i. and increase between day 7 and 28 p.i. (Fig. S1, B and C).

**Figure 1.**
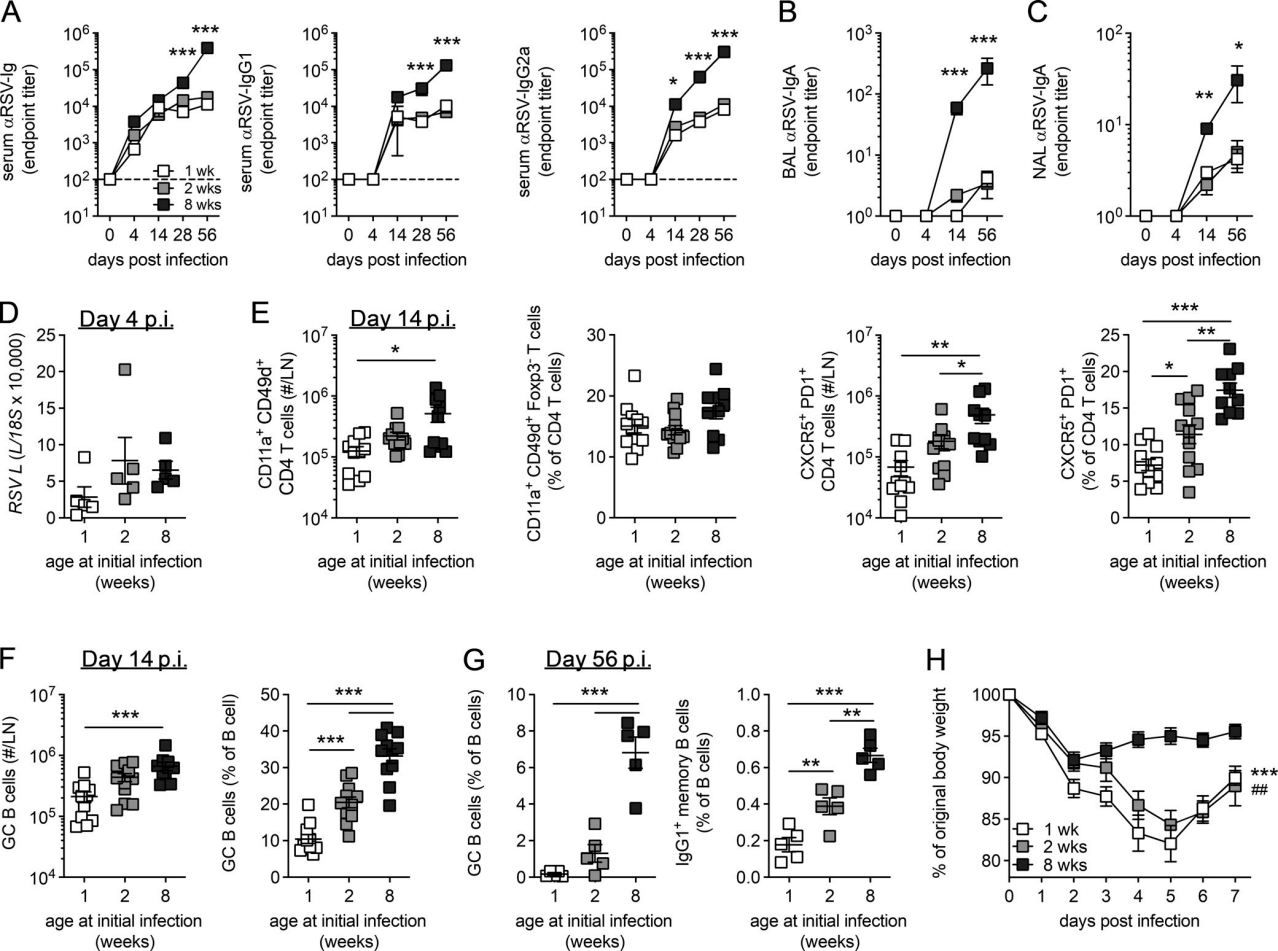
**Early-life RSV infection is associated with reduced GC activity.** 1-, 2-, and 8-wk-old BALB/c mice were infected with 2.5 × 10^5^ ffu RSV A2 i.n. **(A–C)** Endpoint titers of RSV-specific Ig, IgG1, and IgG2a in the serum (A) and RSV-specific IgA in the bronchoalveolar lavage (BAL; B) and nasal lavage (NAL; C) were determined by ELISA. **(D)** RSV *L* relative to *18S* in lung tissue measured by RT-qPCR at day 4 p.i. **(E and F)** The number and frequency of antigen-experienced CD11a^+^CD49d^+^Foxp3^−^ CD4 T cells (E) and CXCR5^+^PD1^+^ T_FH_ and B cells and GC B cells (F) in the lung dLNs were determined by flow cytometry at day 14 p.i. **(G)** The frequency of T_FH_, GC B cells, and IgG1^+^ memory (IgD^−^GL7^−^CD38^+^IgG1^+^) B cells in the LNs was determined by flow at 56 d p.i. **(H)** Mice were rechallenged with 1 × 10^6^ ffu RSV A2 i.n. 8 wk after primary infection, and weight loss was monitored. Area under the curve at 1 wk (*) and 2 wk (#) compared with 8 wk. Data are shown with mean ± SEM. Data in A–C, E, and H are *n* = 10–12 mice per time point and are representative of two independent experiments. Data in D and G are from *n* = 5 mice from one experiment and are representative of two independent experiments presented as *, P < 0.05; ** or ##, P < 0.01; and ***, P < 0.001.

To investigate the role of age on humoral responses 1-, 2-, and 8-wk-old mice were infected i.n. with the same initial dose of RSV. Age had a significant impact on the generation of circulating RSV-specific Ig, with IgG (Fig. 1 A) and locally secreted IgA (Fig. 1, B and C) significantly lower in mice infected in the first 2 wk of life, with no significant difference seen between mice infected at either 1 or 2 wk of age. Expression of RSV *L* gene in the lungs at day 4 p.i., the peak of RSV viral replication in mice, was similar among the different age groups (Fig. 1 D). Mice infected at 1, but not 2, wk of age had fewer CD4^+^ T cells in their LNs at day 14 p.i. compared with adults (Fig. S1 D). Likewise total antigen-experienced CD4^+^ T cells (Foxp3^−^CD11a^+^CD49d^+^) and regulatory Foxp3^+^ CD4^+^ T (T reg) cells in the LNs p.i. were lower in 1-wk-old mice compared with 8-wk-old mice, although proportions were similar (Fig. 1 E and Fig. S1 D). Both the number and proportion of these antigen-experienced cells that differentiated into CXCR5^+^ PD1^+^ T_FH_ was significantly higher when mice were infected as adults, with the proportion that differentiated into T_FH_ increasing progressively with age (Fig. 1 E). In contrast, the number and proportion of Foxp3^+^ T follicular regulatory cells (T_FR_) in the LNs were similar among the different ages of mice (Fig. S1 D). A similar proportion of cytokine-secreting (IFN-γ, IL-21, or IL-4) LN-derived CD4^+^ T cells was observed across all ages after virus-specific peptide stimulation, although there was a trend toward decreased IL-21 production in 1-wk-old mice (Fig. S1 E). A similar pattern was seen in the B cell response, with fewer total B cells in the LN from mice infected at 1 wk of age (Fig. S1 D), and the number and proportion of GC B cells (IgD^−^CD38^−^IgM^−^GL7^+^) increased progressively with age at initial infection (Fig. 1 F). In line with previous studies (Ruckwardt et al., 2011), the proportion of virus-specific CD8^+^ T cells present in the lungs in early life was greatly reduced compared with 8-wk-old mice, although proportions were similar in the LNs (Fig. S1 F).

GCs were readily detectable in both the LN and spleen of mice infected at 8 wk of age, while few were observed in mice infected at 1 wk of age (Fig. S1 G). Mice infected in the neonatal period lacked sustained GC activity, with fewer T_FH_, GC B cells, or IgG1^+^ memory B cells (IgD^−^IgG1^+^GL7^−^CD38^+^) 8 wk after initial infection compared with mice infected at 8 wk of age (Fig. 1 G). Concomitant with the reduced antibody-mediated immunity, mice initially infected at 1 or 2 wk of age showed limited protection from reinfection, suffering rapid weight loss that peaked at day 5 p.i. (Fig. 1 H). These data demonstrate a significant defect in the ability of neonatal mice to mount protective antibody-mediated immunity in response to RSV when compared with adult mice. This was associated with a selective failure in the ability of activated CD4^+^ T cells to form T_FH_ and B cells to differentiate into GC B cells.

### T_FH_ are critical in regulating the outcome of secondary RSV infection

Early-life infection was associated with a failure to generate T cell–dependent antibody responses in the secondary lymphoid compartment and enhanced disease during RSV reinfection. Since T_FH_ are essential in generating GC-dependent antibody and T_FH_ formation was found to be defective in early life, the importance of T_FH_ in protective immunity against RSV was examined.

**Figure 2.**
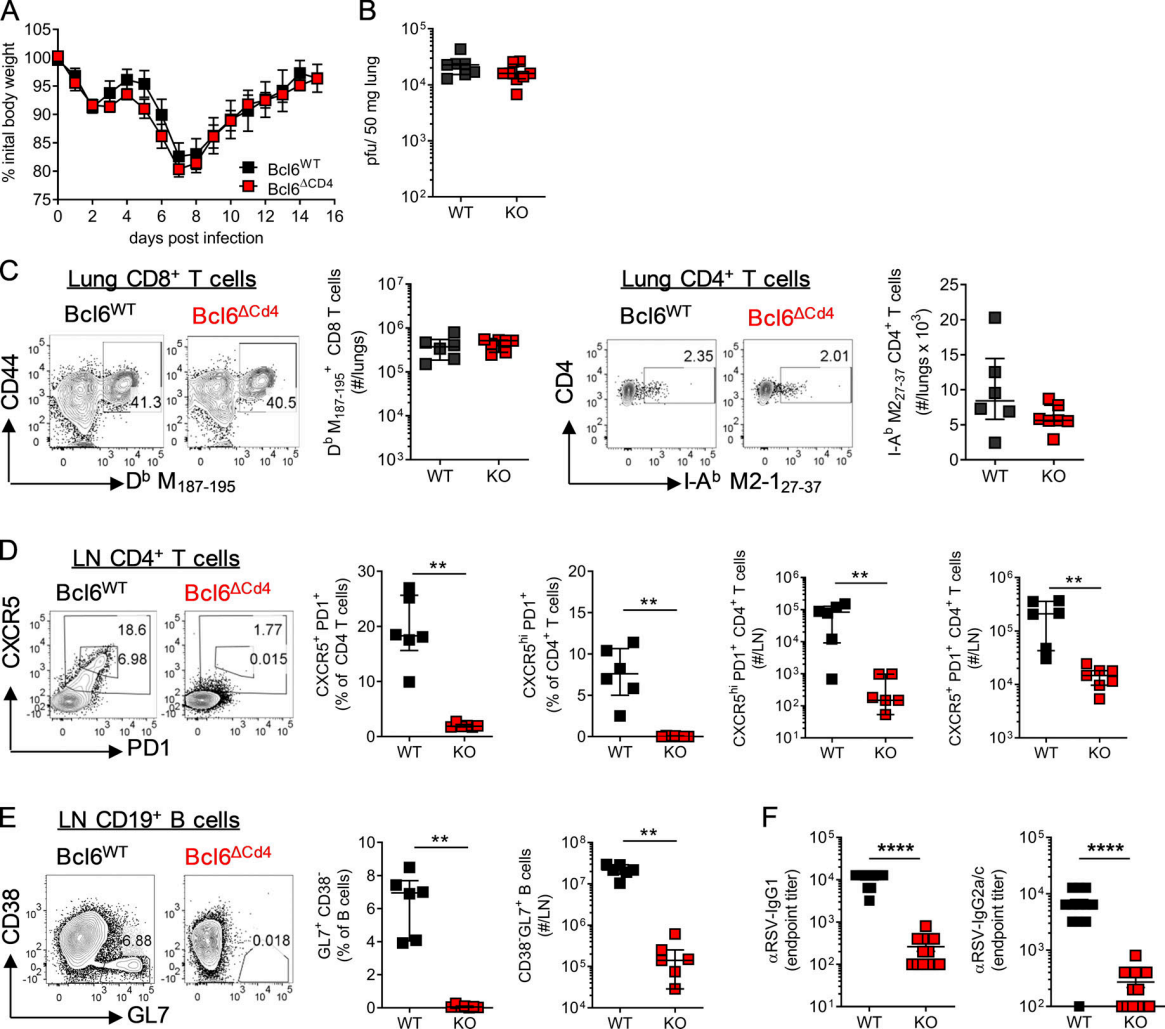
**T cell expression of Bcl6 is essential for humoral immunity but does not affect disease severity after primary RSV infection.** 7- to 8-wk-old WT (*Bcl6^fl/fl^*) or T_FH_-deficient KO (*Cd4*cre *Bcl6^fl/fl^*) mice were infected with 1 × 10^6^ ffu RSV A2 i.n. **(A)** Weight was monitored throughout infection. **(B)** RSV viral load in the lungs at day 4 p.i. as detected by RT-qPCR. **(C)** Virus-specific CD8 and CD4 T cells were analyzed in the lungs at day 14 p.i. **(D and E)** LN T_FH_ (CXCR5^+^ PD1^+^) CD4 T cells (D) and GC (CD38^−^GL7^+^) B cells (E) were analyzed by flow cytometry. **(F)** RSV-specific IgG1 and IgG2a were measured in the serum at day 14 p.i. Data in A and F represent *n* = 12–13 mice per group from two independent experiments. Data in B–E are from *n* ≥ 5 mice per group and are representative of two independent experiments. Data are shown as mean ± SEM. **, P < 0.01; ****, P < 0.0001.

Bcl6 is essential for the differentiation of T_FH_ in vivo (Johnston et al., 2009; Nurieva et al., 2009; Yu et al., 2009). Adult *Cd4*-cre *Bcl6*^fl/fl^ (*Bcl6*^ΔCD4^) mice, which are unable to generate T_FH_, and littermate controls (*Bcl6*^WT^) were infected with RSV, and disease progression was monitored. Disease severity as measured by weight loss was unaltered, peaking at day 7 p.i. in both *Bcl6*^WT^ and *Bcl6*^ΔCD4^ mice (Fig. 2 A). Peak viral load at day 4 p.i. was comparable between WT and *Bcl6*^ΔCD4^ mice (Fig. 2 B), as were virus-specific H2-D^b^ M_187–195_ CD8^+^ and I-A^b^ M2-1_27–37_ CD4^+^ T cell responses in the lungs (Fig. 2 C). CXCR5^+^ PD1^+^ T_FH_ and CXCR5^hi^ PD1^+^ T_FH_ populations, which are thought to be associated with the GC itself, were limited in the lung dLNs of *Bcl6*^ΔCD4^ mice at day 14 after RSV infection but abundant in *Bcl6*^WT^ controls (Fig. 2 D). T cell–specific deletion of Bcl6 also resulted in a several-log-fold reduction in the number of GC B cells in the LNs (Fig. 2 E). RSV-specific IgG1 and IgG2c were significantly lower in *Bcl6*^ΔCD4^ mice (Fig. 2 F).

**Figure 3.**
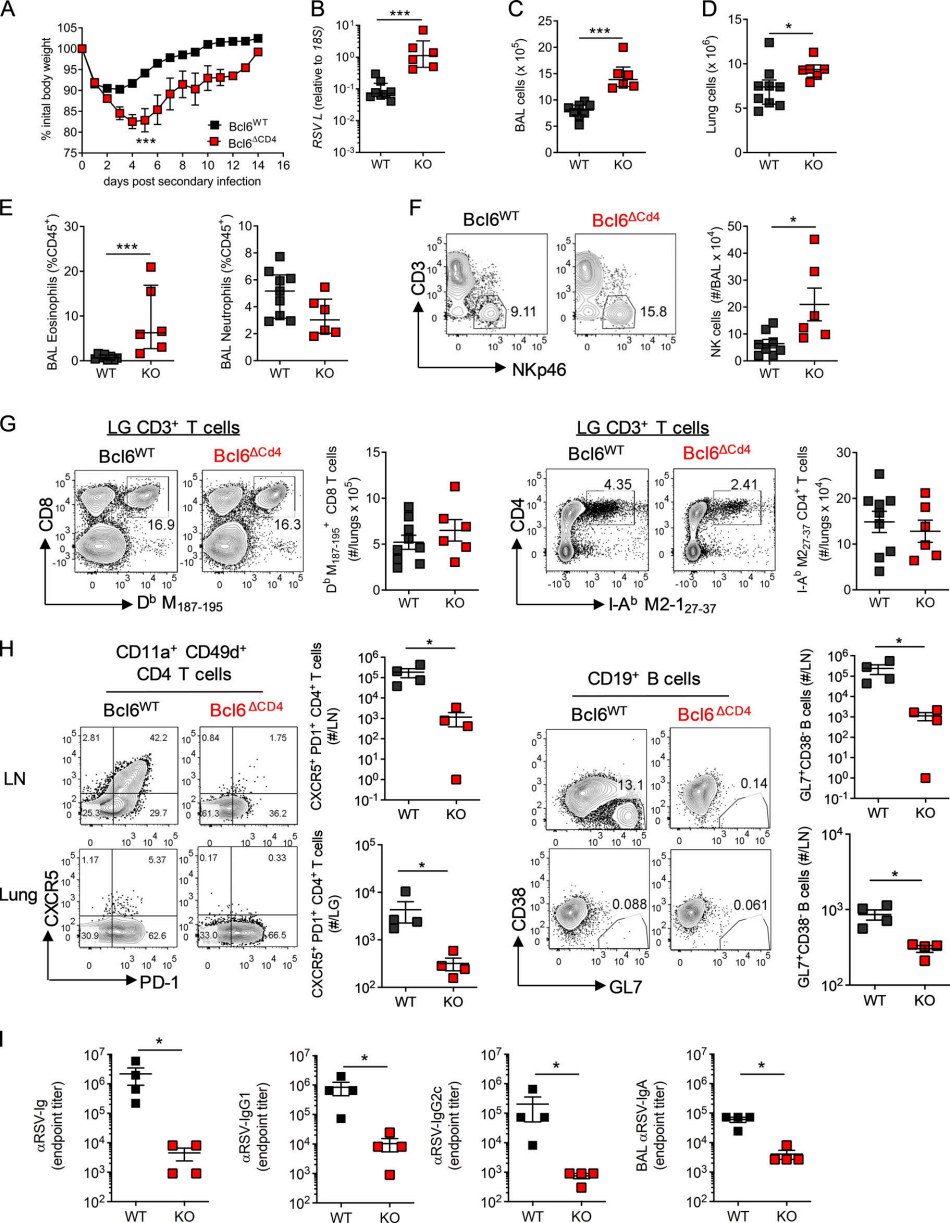
**Impaired humoral immunity limits protection from RSV reinfection.** 7- to 8-wk-old WT (*Bcl6^fl/fl^*) or T_FH_-deficient KO (*Cd4*cre *Bcl6^fl/fl^*) mice were infected with 1 × 10^6^ ffu RSV A2 i.n. and 4 wk later reinfected with 1 × 10^6^ ffu RSV A2 i.n. **(A)** Weight change was monitored throughout infection. **(B)** RSV viral load in the lungs at day 4 p.i. detected by RT-qPCR. **(C and D)** Airway and lung cell numbers. **(E and F)** Proportion of eosinophils and neutrophils (E) and number of NK cells in the airways at day 4 p.i. as measured by flow cytometry (F). **(G)** Virus-specific CD8 and CD4 T cells from the lungs were analyzed by flow cytometry at day 14 p.i. **(H)** T_FH_ and GC B cell numbers were determined in the lungs and LN by flow cytometry, and total numbers were enumerated. **(I)** RSV-specific Ig, IgG_1_, and IgG_2a_ in the serum and IgA in the airways were determined by ELISA. Data are representative of *n* = 12–13 mice per group from two independent experiments. Data are shown with mean ± SEM. *, P < 0.05; and ***, P < 0.001.

While Bcl6 expression in T cells appeared to be redundant in determining the outcome of primary RSV infection, it did impact the antibody response. We therefore investigated the effect on disease when mice were reinfected with the same dose of RSV 5 wk after primary infection. *Bcl6*^ΔCD4^ mice had significantly greater weight loss and delayed recovery compared with *Bcl6*^WT^ littermate controls (Fig. 3 A). This was associated with an increased viral load at day 4 p.i. (Fig. 3 B) and increased cell numbers in the airways and lungs (Fig. 3, C and D). Eosinophil, but not neutrophil, proportions were increased in the airways of *Bcl6*^ΔCD4^ mice compared with *Bcl6*^WT^ controls (Fig. 3 E), as were natural killer (NK) cell numbers (Fig. 3 F).

**Figure S2.**
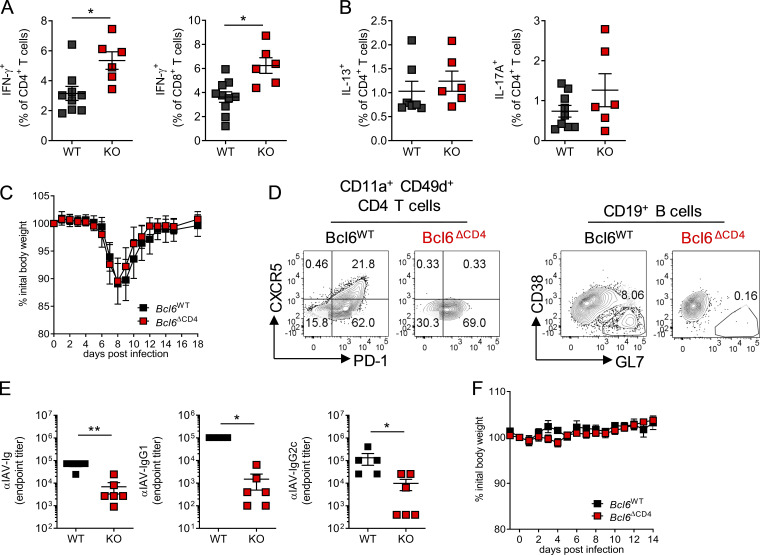
**Bcl6 deficiency in T cells during IAV primary infection and secondary infection reduces antibody responses, but does not affect weight loss. **7- to 8-wk-old WT (*Bcl6^fl/fl^*) or T_FH_-deficient KO (*Cd4*cre *Bcl6^fl/fl^*) mice were infected with 10^6^ ffu RSV A2 i.n. **(A and B)** Virus-specific CD4 and CD8 T cells were stimulated ex vivo with RSV peptides to induce cytokine production and analyzed by flow cytometry. **(C–F)** 7- to 8-wk-old WT (*Bcl6^fl/fl^*) or T_FH_-deficient KO (*Cd4*cre *Bcl6^fl/fl^*) mice were infected with 40 PFU IAV PR8 and weighed daily to monitor disease severity. **(D)** T_FH_ and GC B cell populations were analyzed by flow cytometry. **(E)** IAV-specific Ig, IgG_1_, and IgG_2c_ in the serum as determined by ELISA. **(F)** WT or T_FH_-deficient KO mice were rechallenged with 40 PFU IAV PR8 8 wk after primary challenge and weighed daily. Data are from *n* ≥ 5 mice per group and representative of two independent experiments. Data are shown with mean ± SEM. *, P < 0.05; and **, P < 0.01.

The magnitude of the virus-specific CD8 and CD4^+^ T cell response in the lungs was similar in *Bcl6*^WT^ and *Bcl6*^ΔCD4^ mice (Fig. 3 G), but a greater proportion of virus-specific CD4^+^ and CD8^+^ T cells in the lungs of *Bcl6*^ΔCD4^ mice produced IFN-γ^+^ compared with controls (Fig. S2 A). The frequency of IL-17A^+^ and IL-13^+^ CD4^+^ T cells remained similar (Fig. S2 B). Despite the increased inflammation and disease severity in mice lacking Bcl6^+^ CD4^+^ T cells, they failed to develop either T_FH_ or GC B cells in the LNs or lungs (Fig. 3 H) and had substantially lower virus-specific antibodies even after reinfection (Fig. 3 I), reaffirming the importance of T_FH_ in promoting humoral immunity.

Infection of *Bcl6*^WT^ and *Bcl6*^ΔCD4^ mice with a different respiratory virus, H1N1 influenza A virus (IAV) strain PR8, resulted in similar disease severity on primary infection (Fig. S2 C). As with RSV, *Bcl6*^ΔCD4^ mice failed to develop GC B cells or T_FH_ cells and had significantly reduced virus-specific Ig, IgG1, and IgG2c responses (Fig. S2, D and E). Despite this, and unlike RSV-infected mice, IAV-infected mice did not lose weight during reinfection with the same strain of IAV 4 wk later (Fig. S2 F), fitting with the observation that in IAV infection, other pathways, including tissue-resident T cells and non-T_FH_–dependent generation of antibody, may be sufficient to protect against reinfection (Miyauchi et al., 2016; Wu et al., 2014).

These data demonstrate that T_FH_ are essential in promoting GC formation and antibody responses in response to respiratory virus infection but did not alter CD8^+^ T cell responses. Further, GC B cell activity was associated with protecting RSV-infected, but not IAV-infected, mice from disease on reinfection.

### Disease resolution after RSV infection of neonatally primed mice is T_FH_ dependent

**Figure S3.**
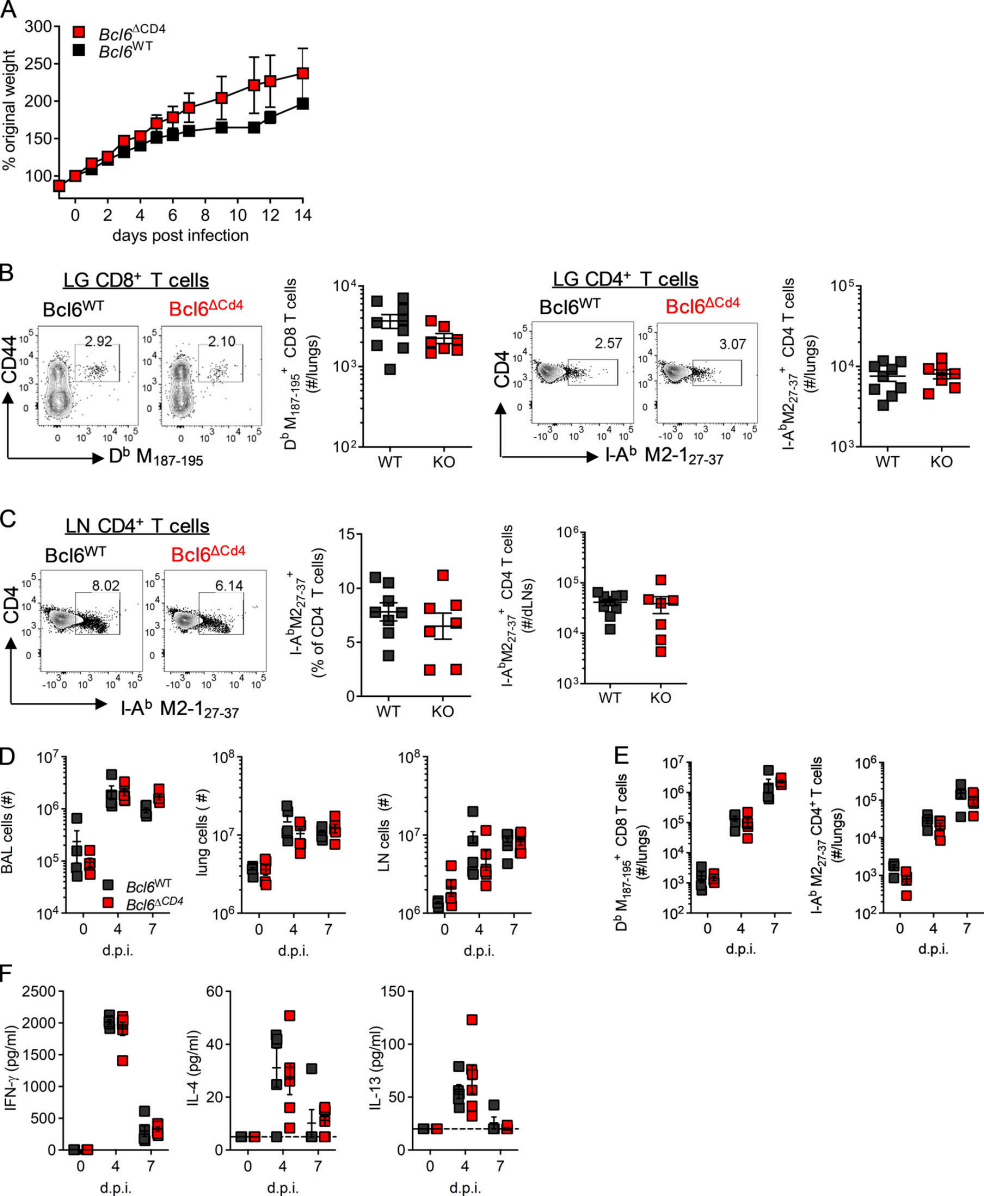
**BCL6 deficiency in early life does not affect the acute immune response.** WT (*Bcl6^fl/fl^*) or T_FH_-deficient KO (*Cd4*cre *Bcl6^fl/fl^*) mice were infected as neonates (7 d old) with 2.5 × 10^5^ ffu RSV A2 i.n. **(A)** Mice infected as neonates were weighed daily. **(B and C)** Virus-specific T cell populations were analyzed in the lungs (B) and LN (C) of infected neonates at day 14 p.i. **(D–F)** Mice were reinfected 8 wk later with 8 × 10^5^ ffu RSV A2 i.n. **(D)** Airway, lung, and LN cells before (day 0) and after (days 4 and 7) reinfection. **(E)** Virus-specific T cell populations were analyzed by flow cytometry at all time points. **(F)** Cytokine concentrations in the airways was measured by ELISA. Data are from *n* ≥ 4 mice per group and representative of two independent experiments. Data are shown with mean ± SEM.

**Figure 4.**
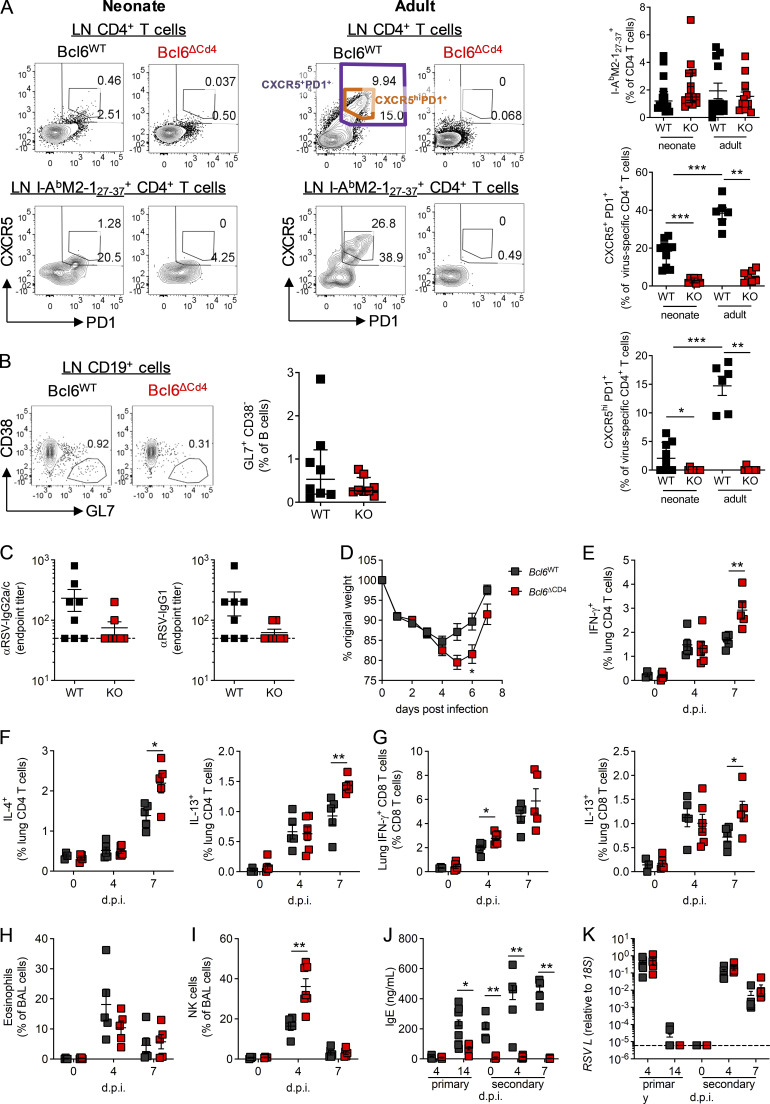
**Early-life immune responses to RSV are T_FH_ independent.** WT (*Bcl6^fl/fl^*) or T_FH_-deficient KO (*Cd4*cre *Bcl6^fl/fl^*) mice were infected as neonates (7 d old) with 2.5 × 10^5^ ffu or as adults (7–8 wk old) with 1 × 10^6^ ffu RSV A2 i.n. **(A)** Virus-specific CD4 T cells and T_FH_ populations in the LNs of mice infected as neonates or adults were analyzed by flow cytometry at day 14 p.i. Gating for CXCR5^+^PD1^+^ (purple line) and CXCR5^hi^PD1^+^ (orange line) are highlighted. **(B)** LN GC B cells were analyzed by flow cytometry at day 14 p.i. in mice infected as neonates. **(C)** Virus-specific serum IgG1 and IgG2c was measured by ELISA. **(D)** Mice infected as neonates were reinfected 8 wk later with 8 × 10^5^ ffu RSV A2 i.n. and weighed daily. **(E–G)** Virus-specific IFN-γ (E) and IL-4 and IL-13 (F) production by CD4 and CD8 T cells (G) was analyzed by flow cytometry before reinfection and at days 4 and 7 p.i. **(H and I)** Airway eosinophils (H) and NK cells (I) were analyzed by flow cytometry. **(J)** Serum IgE was quantified during primary and secondary infections by ELISA. **(K)** Lung viral load was detected by RT-qPCR at all time points. Data are from *n* = 4–9 mice per time point and representative of two independent experiments. Data are shown with mean ± SEM. *, P < 0.05; **, P < 0.01; and ***, P < 0.001.

To determine the role of Bcl6 expression by T cells on the outcome of RSV infection during early life, *Bcl6*^WT^ and *Bcl6*^ΔCD4^ mice were infected with RSV at day 7 of life. Weight gain was similar in both groups following infection (Fig. S3 A). At day 14 p.i., H2-D^b^ M_187–195_^+^ CD8^+^ T cells were identifiable in the lungs of both *Bcl6*^WT^ and *Bcl6*^ΔCD4^ mice, although at a much lower proportion and frequency than was observed after infection of adult animals, fitting with previous observations of an altered, and reduced, CD8^+^ T cell hierarchy in neonatally infected mice (Ruckwardt et al., 2011; Fig. 2 C and Fig. S3 B). The proportion of CD4^+^ T cells specific for I-A^b^M2-1_27–37_^+^ in the lungs and dLNs was similar to that seen 14 d p.i. in adults and did not change in T_FH_-deficient mice (Fig. 4 A; and Fig. S3, B and C).

T_FH_ differentiation, in both polyclonal and I-A^b^M2-1_27–37_^+^ CD4^+^ T cells, was impaired in neonates compared with adults, with GC-localized CXCR5^hi^ PD1^+^ T_FH_ being particularly lower (Fig. 4 A). As in adults, Bcl6 deficiency prevented T_FH_ differentiation entirely (Fig. 4 A). Fitting with this, GC B cells were low in frequency after early-life infection, with no significant difference seen between WT littermates and *Bcl6*^ΔCD4^ mice (Fig. 4 B). Virus-specific antibody was also limited after early-life infection, with no difference between WT and KO mice (Fig. 4 C).

Reinfection of neonatally primed mice 8 wk later resulted in rapid (within 24 h) weight loss that peaked around day 5 p.i. Weight loss on reinfection was greater in *Bcl6*^ΔCD4^ mice than littermate controls, and recovery was delayed (Fig. 4 D). Cell counts in the airways, lungs, and LNs before and after reinfection were comparable between the two groups (Fig. S3 D). The enhanced weight loss and cellular inflammation seen on reinfection of neonatally primed mice is associated with both a T helper type 2 (Th2) skewed immune response not normally seen in adult mice, and exacerbated NK and CD8 T cell responses (Culley et al., 2002; Harker et al., 2014; Tregoning et al., 2008). Virus-specific CD4^+^ and CD8^+^ T cell numbers in the lungs after reinfection were similar between WT and Bcl6-deficent mice (Fig. S3 E). After reinfection, WT mice had lower Th1 (IFN-γ^+^), Th2 (IL-4^+^ and IL-13^+^), and T cytotoxic type 2 (IL-13^+^ CD8^+^) cell proportions at day 7, and IFN-γ^+^ CD8 T cells were slightly increased in T_FH_-deficient animals at day 4 p.i. (Fig. 4, E–G). IFN-γ, IL-4, and IL-13 concentrations in the airways were similar between WT and *Bcl6*^ΔCD4^ mice (Fig. S3 F), as was eosinophilia (Fig. 4 H); however, airway NK cell frequencies were elevated in *Bcl6*^ΔCD4^ mice compared with controls (Fig. 4 I). IgE was detectable in the sera of control mice after primary neonatal infection and elevated on reinfection but was largely absent in T_FH_-deficient mice (Fig. 4 J). Viral loads during both primary and secondary infection of neonates were similar between control and *Bcl6*^ΔCD4^ mice (Fig. 4 K).

The humoral response to neonatal RSV infection was significantly lower than in adults; this was associated with a failure of virus-specific CD4^+^ T cells to differentiate into T_FH_. Genetic ablation of T_FH_ during neonatal infection had no further impact on the antibody response, indicating their absence is a critical limiting factor, although *Bcl6*^ΔCD4^ mice had a more prolonged disease course on viral reinfection.

### IFN-γ and IL-2 signaling dampen B cell immunity in early life

Inadequate neonatal T_FH_ responses to RSV infection contribute to augmented disease on reinfection; we subsequently investigated why this was the case. In mice, IFN-γ has previously been found to limit antibody responses after early-life RSV infection (Tregoning et al., 2013) and has been shown to limit T_FH_ during severe malarial infection (Ryg-Cornejo et al., 2016). IL-2 signaling through STAT-5 also regulates T_FH_ differentiation in IAV infection (Ballesteros-Tato et al., 2012).

**Figure 5.**
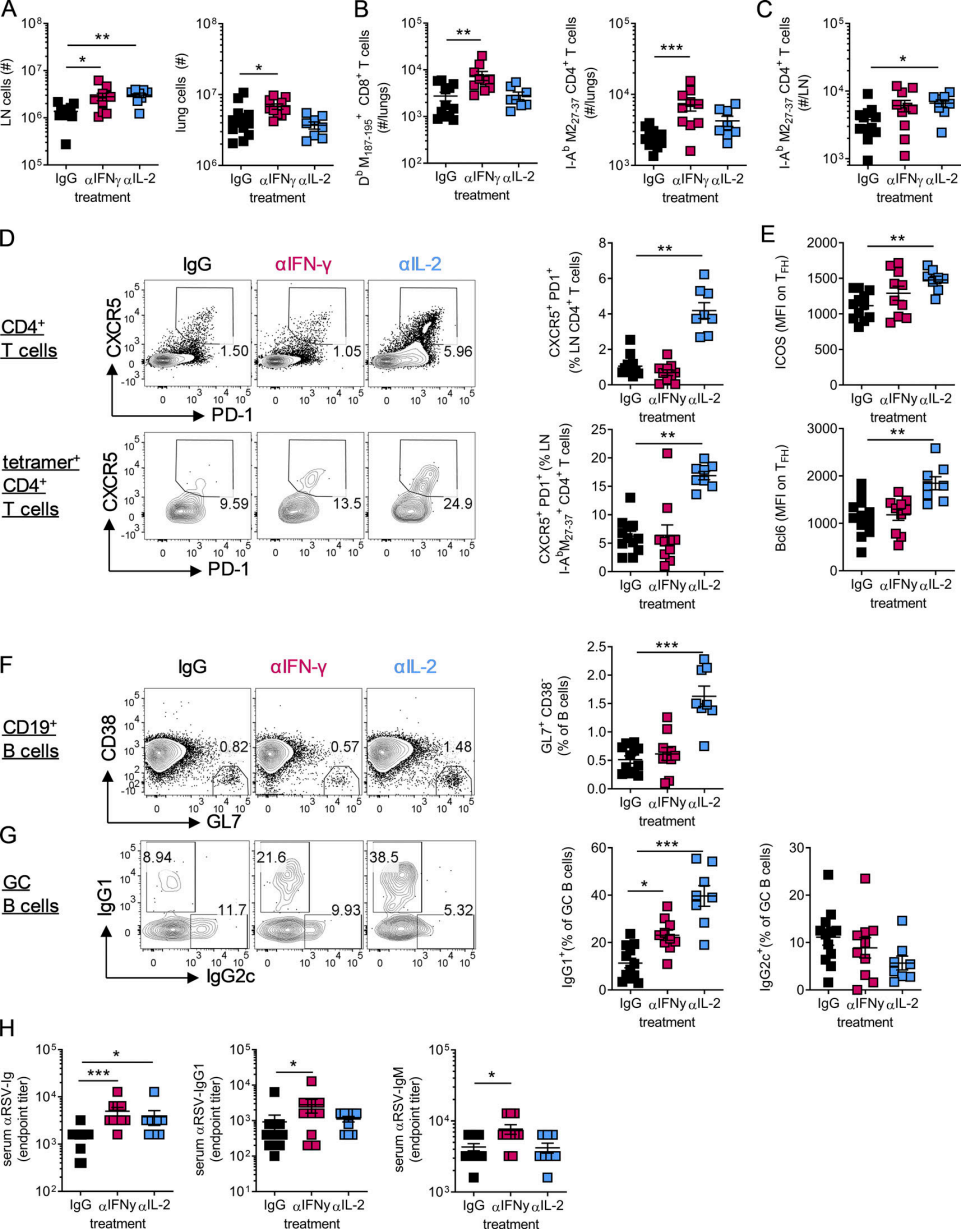
**IL-2 and IFN-γ signaling limit antibody-mediated immunity after early-life infection.** 7-d-old C57B/L6 mice were infected with 2.5 × 10^5^ PFU RSV A2 i.n. and treated with anti-IFN-γ, anti-IL-2, or isotype-matched antibodies i.p. on days −1, 2, and 5 p.i. **(A)** At day 14 p.i., lung and LN cells were counted. **(B and C)** Virus-specific CD4 and CD8 T cell populations in the lung (B) and dLN (C) were analyzed by flow cytometry. **(D–G)** Total and virus-specific T_FH_ and ICOS and Bcl6 expression on total T_FH_ (D and E) and GC (F and G) B cell subsets were analyzed by flow cytometry. **(H)** RSV-specific Ig, IgG1, and IgM were measured by ELISA. Data are from *n* = 8–12 mice per group and representative of two independent experiments. Data are shown with mean ± SEM. *, P < 0.05; **, P < 0.01; and ***, P < 0.001.

**Figure S4.**
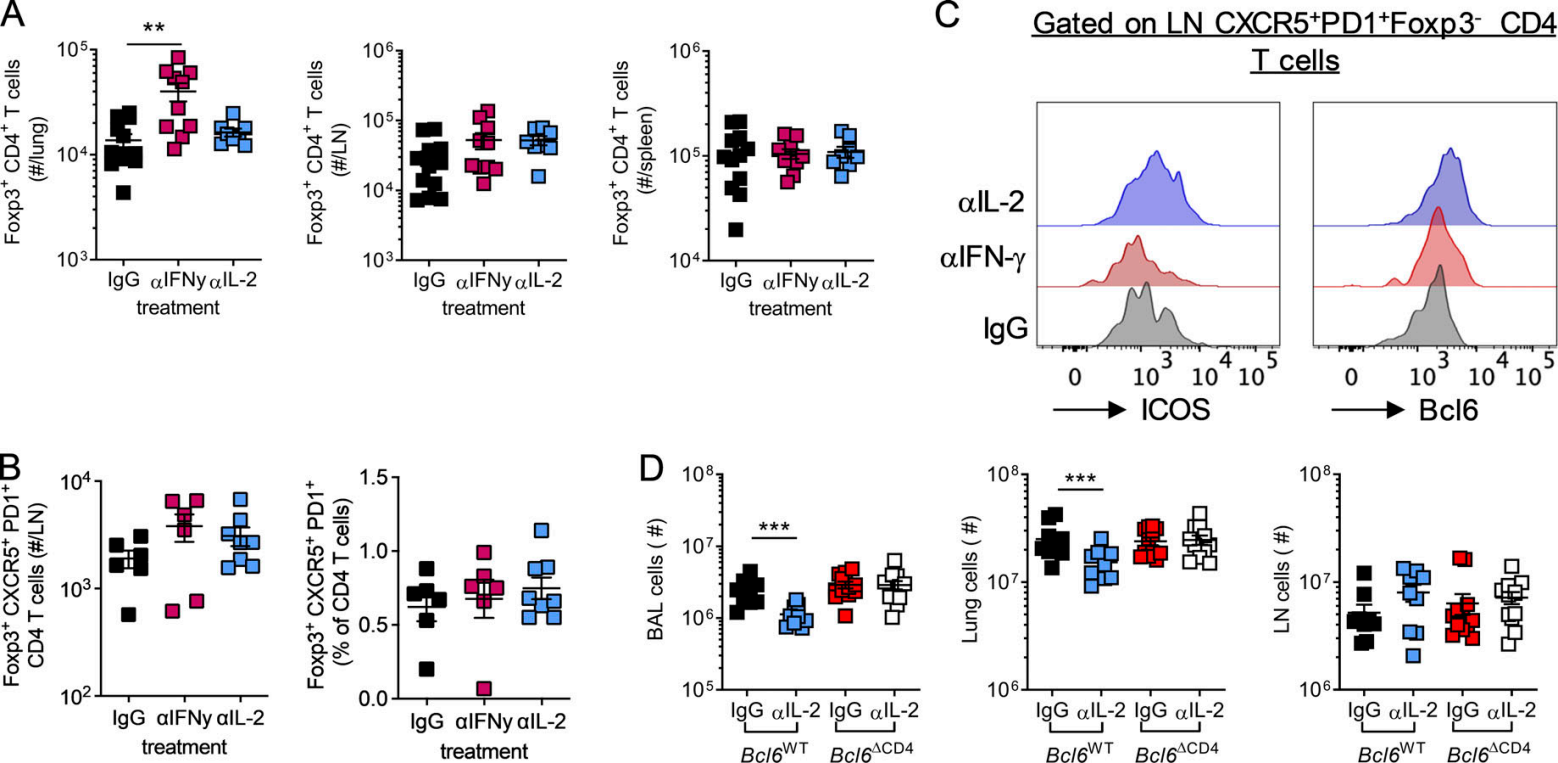
**IL-2 neutralization does not limit T reg cell numbers in early-life RSV infection but limits inflammation on reinfection as adults. **Mice were infected with 2.5 × 10^5^ ffu RSV A2 at 7 d of age and treated with anti-IFN-γ, anti-IL-2, or isotype control i.p.; mice were sacrificed at day 14 p.i. **(A)** Foxp3^+^ T reg cells were enumerated in the lung, LNs, and spleen. **(B)** The number and frequency of Foxp3^+^CXCR5^+^PD1^+^ CD4 T cells in the lung dLNs. **(C)** ICOS and Bcl6 expression by LN T_FH_ was determined by flow cytometry, and representative histograms are shown. **(D)** Total airway, lung, and LN cell counts at day 7 after reinfection with 8 × 10^5^ PFU RSV A2 i.n. in mice initially infected with 2.5 × 10^5^ PFU RSV A2 i.n. as neonates. Data represent two independent experiments with *n* = 8–12 mice per group. Data are shown with mean ± SEM. **, P < 0.01; and ***, P < 0.001.

Anti-IFN-γ and anti-IL-2 treatment resulted in distinct outcomes on the immune response to RSV. At day 14 p.i., both treatments increased cell numbers in the LNs compared with isotype control–treated neonates, but only anti-IFN-γ increased cell numbers in the lungs (Fig. 5 A). Anti-IFN-γ treatment resulted in expanded H2-D^b^ M_187–195_^+^–specific CD8^+^ and I-A^b^ M2-1_27–37_^+^ CD4^+^ T cell responses in the lungs compared with isotype-treated controls, changes not seen in the anti-IL-2 group (Fig. 5 B). In the LNs, anti-IL-2, but not anti-IFN-γ, treatment resulted in increased numbers of I-A^b^M2-1_27–37_^+^ CD4^+^ T cells (Fig. 5 C). IL-2 signaling is important in the formation and function of T reg cells (reviewed in Malek and Castro, 2010); however, in the current study, transient neutralization of IL-2 did not alter the number of Foxp3^+^ T reg cells in the lungs, LNs, or spleen, while anti-IFN-γ treatment increased T reg cell numbers in the lungs, in line with its effects on total lung cell count (Fig. S4, A and B).

Depleting IL-2 during neonatal RSV did, however, increase the proportion of CD4^+^ T cells and I-A^b^M2-1_27–37_^+^ CD4^+^ T cells that were CXCR5^+^ PD1^+^ (Fig. 5 D). It also altered the phenotype of the T_FH_, increasing the expression of both Bcl6 and ICOS compared with isotype controls (Fig. 5 E and Fig. S4 C). Concomitant with this, the proportion of GL7^+^CD38^−^ GC B cells in the LNs increased after anti-IL-2 treatment (Fig. 5 F). The frequency of GC B cells that were IgG1^+^ was also significantly increased after both anti-IL-2 and anti-IFN-γ treatment compared with control mice, while IgG2c expression was slightly reduced (Fig. 5 G). Neutralizing either IFN-γ or IL-2 during neonatal RSV infection resulted in increased RSV-specific Ig at day 14 p.i., with anti-IFN-γ also resulting in significantly increased RSV-specific IgM and IgG1 (Fig. 5 H). Together, these data showed that both IFN-γ and IL-2 signaling suppress IgG1 responses during early-life viral infection, with IFN-γ broadly limiting immune responses and IL-2 appearing to specifically attenuate the formation of T_FH_ and GC B cells.

### IL-2–dependent suppression of T_FH_ prevents protective immunity in early life

**Figure 6.**
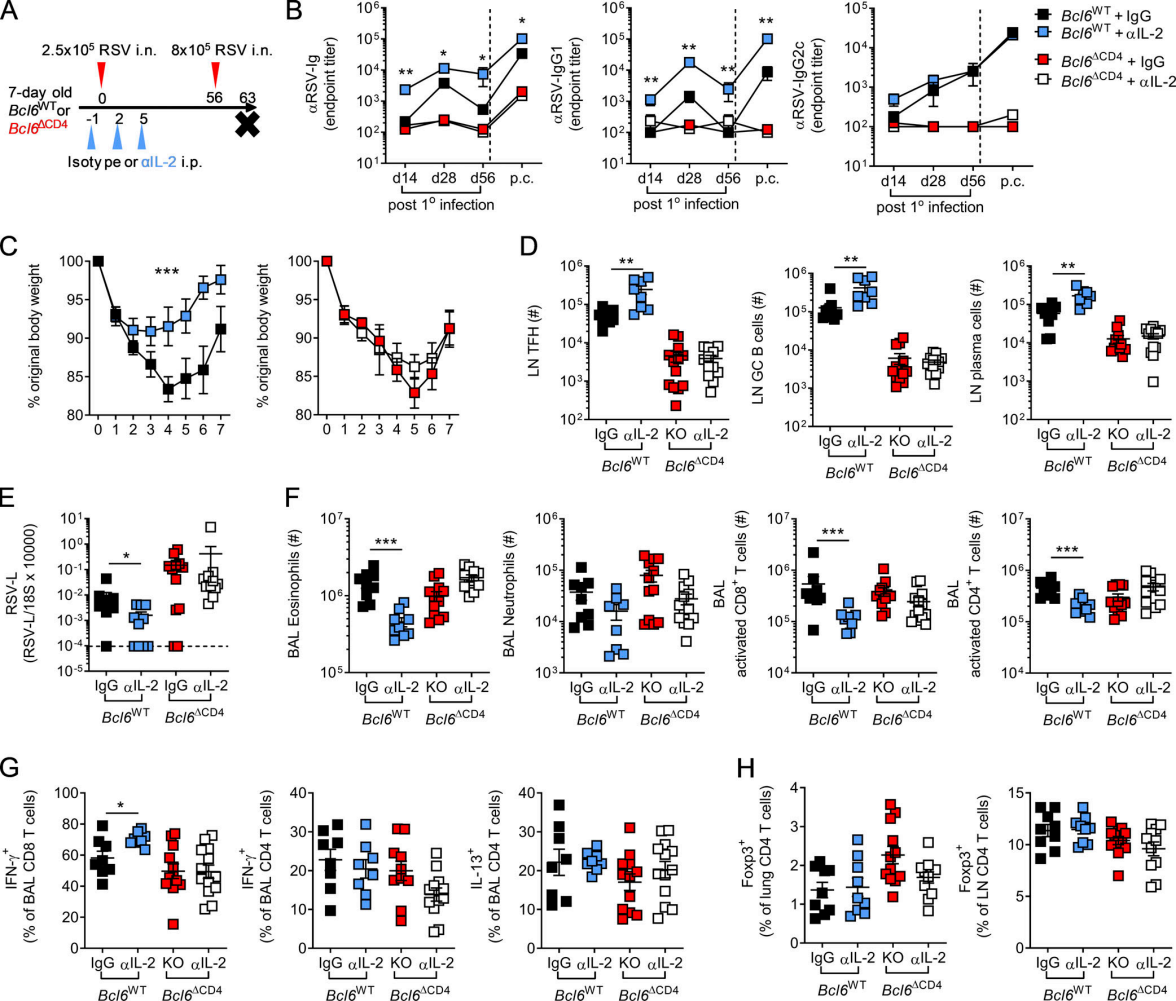
**Neutralizing IL-2 in early life enhances protective immunity to secondary infection in a T_FH_-dependent manner.** 7-d-old *Bcl6*^WT^ or *Bcl6*^ΔCD4^ mice were infected with 2.5 × 10^5^ PFU RSV A2 i.n. and treated with either anti-IL-2 or isotype-matched antibodies i.p. on days −1, 2, and 5 p.i. On day 56 p.i., they were then reinfected with 8 × 10^5^ PFU RSV A2 i.n. **(A)** Experimental design. **(B)** RSV-specific serum Ig, IgG1, and IgG2c were measured by ELISA. **(C)** Weight change on reinfection. **(D)** At day 7 after challenge, the number of T_FH_, GC B cells, and CD138^+^CD3^−^ plasma cells were enumerated in the LNs by flow cytometry. **(E and F)** *RSV L* expression in the lungs was determined by RT-qPCR (E), and the number of airway eosinophils, neutrophils, and activated (CD44^+^PD1^+^) CD8 and CD4 T cells was analyzed by flow cytometry (F). **(G)** Following polyclonal stimulation, the frequency of airway CD8 and CD4 T cells producing IFN-γ or IL-13 was measured. **(H)** Foxp3^+^ T reg cell frequencies in the lungs and LNs were determined by flow cytometry. Data represent two independent experiments with *n* = 8–12 mice per group. Data are shown with mean ± SEM. *, P < 0.05; **, P < 0.01; and ***, P < 0.001.

As blockade of IL-2 signaling in early life enhanced GC reactions, we next examined its effects on long-term antibody-mediated immunity and the role of T_FH_ (experimental design shown in Fig. 6 A). Neutralization of IL-2 during primary neonatal RSV infection resulted in significantly increased RSV-specific Ig and IgG1, but not IgG2c, in the serum of *Bcl6*^WT^ mice both at 4 wk and 8 wk p.i., while no effect was observed in *Bcl6*^ΔCD4^ mice (Fig. 6 B). Following reinfection of neonatally infected mice at 8 wk p.i., RSV-specific antibodies rapidly increased in *Bcl6*^WT^ mice, with those treated with anti-IL-2 in early life still having significantly increased concentrations of total Ig and IgG1 (Fig. 6 B). Blocking IL-2 signaling in early life also attenuated weight loss observed on reinfection of neonatally infected WT mice but did not affect weight loss or recovery in *Bcl6*^ΔCD4^ mice (Fig. 6 C). The number of cells in the airways and lungs on reinfection was also reduced in anti-IL-2–treated WT mice, while LN cell counts were unchanged, but no difference was seen when Bcl6 was absent (Fig. S4 D).

In line with the antibody response, anti-IL-2 treatment resulted in enhanced T_FH_, GC B cell, and CD138^+^ plasma cell numbers in the LNs of WT, but not Bcl6-deficient, animals (Fig. 6 D). Anti-IL-2 treatment significantly reduced viral burden in the lungs of reinfected *Bcl6*^WT^ mice, while no difference was observed in *Bcl6* ^ΔCD4^ mice (Fig. 6 E). Anti-IL-2 treatment of neonatal WT mice also reduced the numbers of eosinophils and CD4^+^ and CD8^+^ T cells in the airways but had no significant effect on neutrophil number (Fig. 6 F). Functionally, there was a slight increase in the IFN-γ production capacity of CD8 T cells from the airways of *Bcl6*^WT^ mice treated with anti-IL-2, but IFN-γ and IL-13 production by CD4^+^ T cells was similar (Fig. 6 G). The proportion of T reg cells in the lungs and the LNs was similar across all groups (Fig. 6 H).

These data support the conclusion that IL-2 suppresses antibody-mediated immunity in early life in a T_FH_-dependent manner. Neutralization of IL-2 during primary infection promotes antibody responses and can improve protection and reduce disease on reinfection.

### Multiple mechanisms contribute to IL-2–dependent suppression of T_FH_ in early life

**Figure 7.**
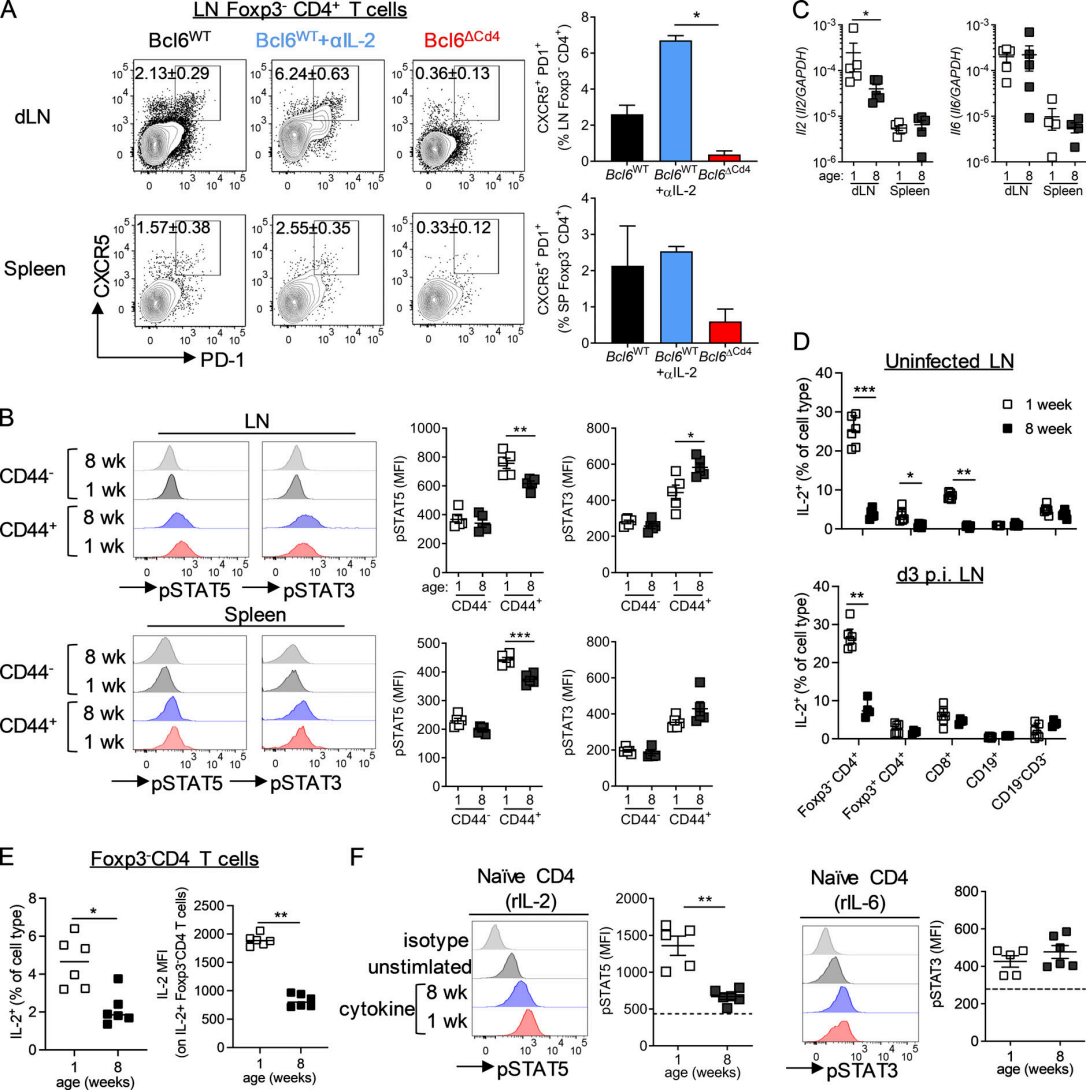
**Enhanced pSTAT5 in early-life CD4 T cells is associated reduced T_FH_ differentiation.** **(A and B)** 7-d-old *Bcl6*^WT^ or *Bcl6*^ΔCD4^ mice were infected with 2.5 × 10^5^ PFU RSV A2 i.n. *Bcl6*^WT^ mice were treated with either anti-IL-2 or isotype-matched antibodies i.p., while *Bcl6*^ΔCD4^ mice received isotype control. T_FH_ were analyzed in the dLNs at day 3 p.i. **(C)** pSTAT3 and pSTAT5 expression measured directly ex vivo in naive (CD44^−^Foxp3^−^GITR^−^) and antigen-experienced (CD44+Foxp3^−^GITR^−^) CD4 T cells in the spleen and dLNs of day 3 p.i. 1- and 8-wk-old C57BL/6 mice. **(D)** *Il2* and *Il6* expression in the spleen of 1-, 2-, and 8-wk-old uninfected C57BL/6 mice. **(D and E)** IL-2 production following polyclonal stimulation in LNs from uninfected or day 3 p.i. RSV-infected 1- and 8-wk-old C57BL/6 mice (D), and (E) the geometric mean fluorescence intensity (MFI) of IL-2 in IL-2^+^ Foxp3^−^ CD4 T cells following polyclonal stimulation (left graph), and the percentage of IL-2^+^ in the presence of brefeldin A alone were measured by flow cytometry. **(F)** CD4 T cells were isolated from the spleens of 1 and 8-wk-old C57BL/6 mice and stimulated for 15 min with rIL-2 or rIL-6. STAT phosphorylation was quantified by flow cytometry. Data are from *n* = 4–6 mice per group and are representative of two independent experiments. Data are shown with mean ± SEM. *, P < 0.05; **, P < 0.01; and ***, P < 0.001.

**Figure S5.**
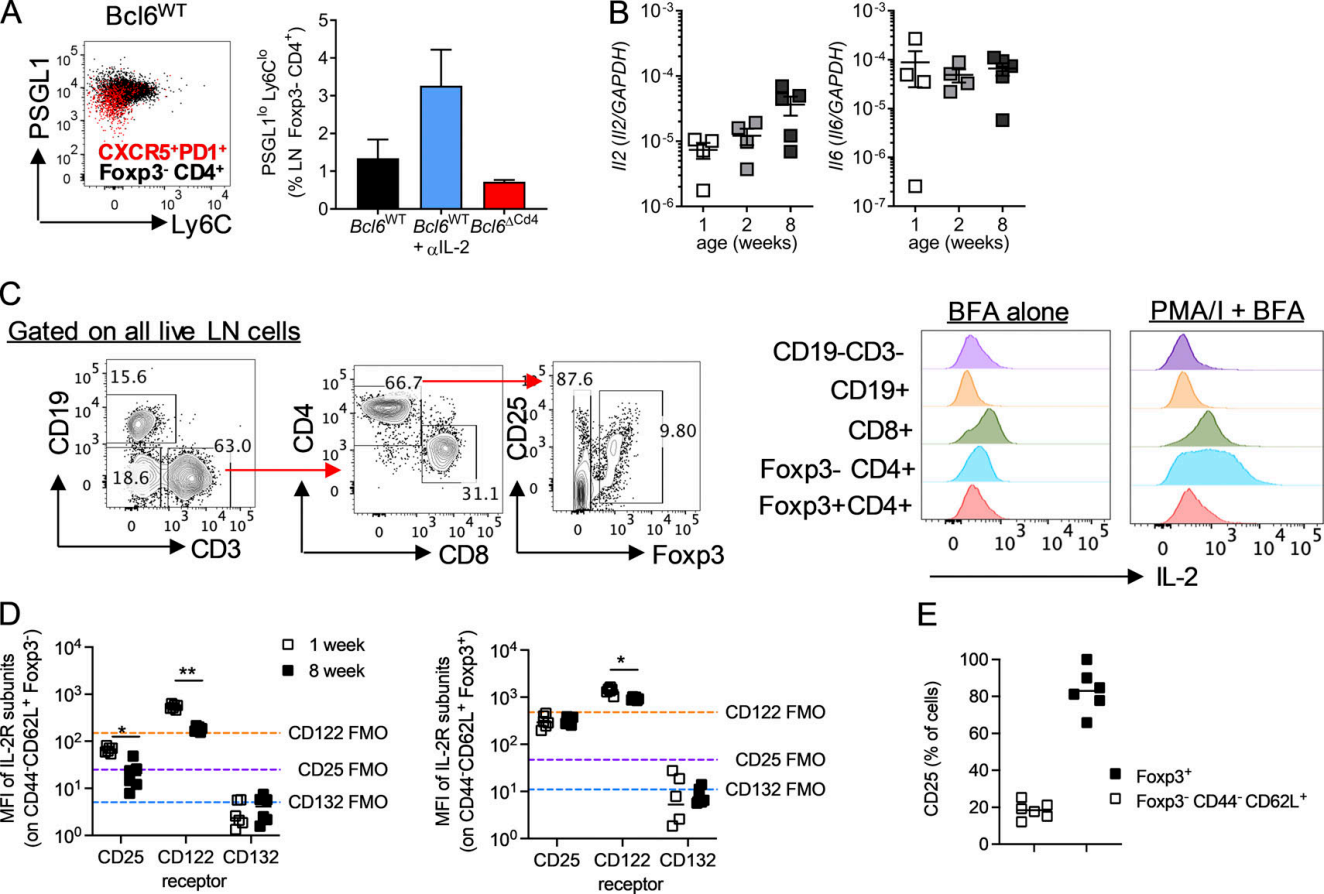
**Assessment of IL-2 and IL-6 production and signaling in secondary lymphoid tissue.** **(A)** Neonatal LN T_FH_ and non-T_FH_, as shown in Fig. 7 A, were assessed for expression of PSGL1 and Ly6C. **(B)** Expression of *Il6* and *Il2* relative to *GAPDH* in the spleen of 1-, 2-, and 8-wk-old uninfected C57B/L6 mice was determined by RT-qPCR. **(C)** IL-2 was measured by flow cytometry in different cell populations from lung dLNs of neonatal C57B/L6 at day 3 after RSV infection. A gating strategy for the cell types and a representative IL-2 histogram following either polyclonal (PMA/I and brefeldin A [BFA]) or unstimulated (brefeldin A alone) are shown. **(D)** CD25, CD122, and CD132 expression by splenic CD44^−^CD62L^+^Foxp3^−^ and Foxp3^+^ CD4 T cells in 1- and 8-wk-old C57BL/6 mice. FMO, fluorescence minus one. **(E)** Percentage of CD25^+^ splenic CD44^−^CD62L^+^Foxp3^−^ and Foxp3^+^ CD4 T cells in 1-wk-old C57BL/6 mice. Data in A and B are from *n* = 4–5 mice per group and are representative of two independent experiments. Data in D and E are from *n* = 6 mice and are representative of two independent experiments. **(C)** Representative gating strategy from an RSV-infected mouse; data are enumerated in Fig. 7, D and E. Data are shown with mean ± SEM. *, P < 0.05; and **, P < 0.01.

We next wanted to explore what blocks the development of T_FH_ cells in early life. IL-2–induced STAT5 signaling has been shown to be capable of suppressing both the differentiation and maintenance of T_FH_ (Ballesteros-Tato et al., 2012; Johnston et al., 2012; Nurieva et al., 2012). Conversely, STAT3, and to a lesser extent STAT1 signaling, through cytokines such as IL-6 can promote T_FH_ differentiation and function (Choi et al., 2013; Harker et al., 2013; Harker et al., 2011; McIlwain et al., 2015; Papillion et al., 2019). T_FH_ differentiation in adult mice occurs within the first few days after acute infection (Choi et al., 2011); 3 d p.i., neonatal WT mice had a population of antigen-experienced, Foxp3^−^ CD4^+^ T cells that had up-regulated CXCR5 and PD1 in both the LNs and the spleen. These cells were not present in Bcl6-deficient animals but were present in increased numbers in the LNs of neonatal WT mice after anti-IL-2 treatment (Fig. 7 A). Supporting their identity as early T_FH_, these cells also had reduced PSGL1 expression and were not Ly6C^+^ (Poholek et al., 2010; Fig. S5 A).

There was a significant increase in phosphorylated STAT5 (pSTAT5) and a decrease in pSTAT3 in neonatal antigen-experienced (CD44^+^Foxp3^−^GITR^−^) CD4^+^ T cells compared with those taken from adults at day 3 p.i., suggesting an environment enriched for STAT5 signaling cytokines in early life (Fig. 7 B). Expression of *Il2* and *Il6* in the spleens of uninfected 1-, 2-, and 8-wk-old mice did not vary depending on age, and gene expression was undetectable in the LNs (Fig. S5 B). However, 3 d after RSV infection, *Il2* transcripts were detectable in the LNs and were significantly higher in 1-wk-old than 8-wk-old mice, while no difference was seen in the spleen (Fig. 7 C). *Il6* expression was similar in both the LNs and spleen of 1- and 8-wk-old mice at 3 d p.i. (Fig. 7 C). Intracellular staining for IL-2 indicated that Foxp3^−^CD4^+^ T cells, alongside CD8^+^ T cells, were the main sources of IL-2 in the LN in response to polyclonal stimulation (Fig. S5 C). IL-2 production by CD4^+^ and CD8^+^ T cells was significantly increased in uninfected mice at 1 wk compared with 8 wk of age, with Foxp3^−^ CD4^+^ T cells becoming the major source in the neonatal LN p.i. (Fig. 7 D). Foxp3 expression is known to prevent IL-2 production by T reg cells (Wu et al., 2006), however in neonatal but not adult mice, some Foxp3^+^ CD4^+^ T cells produced IL-2, although a much lower proportion than the Foxp3^−^ CD4^+^ T cells (Fig. 7 D). This may indicate that Foxp3 expression in T reg cells has not fully stabilized at this age, in line with observations there is a delay in Foxp3 expression in early life (Fontenot et al., 2005). IL-2 production by Foxp3^−^ CD4^+^ T cells remained significantly increased in neonatal mice compared with adults at day 3 after RSV infection, although production by Foxp3^+^ CD4^+^ T cells and CD8 T cells was similar (Fig. 7 D). In the absence of ex vivo stimulation, IL-2 production by naive Foxp3^−^ CD4^+^ T cells was also observed in early life after viral infection, and IL-2^+^ cells expressed more IL-2 per cell than those from adults (Fig. 7 E).

The capacity of naive CD44^−^Foxp3^−^ splenic CD4^+^ T cells from both 1- and 8-wk-old uninfected mice to phosphorylate STAT5 in response to recombinant IL-2 (rIL-2) and STAT3 in response to IL-6 in vitro was examined. While pSTAT3 levels were similar in both age groups after IL-6 stimulation, pSTAT5 levels were significantly heightened in 1-wk-old mice compared with 8-wk-old mice when stimulated with IL-2 (Fig. 7 F). To determine if this increased sensitivity was associated with altered expression of IL-2R proteins we measured expression of the three subunits, CD25 (IL-2Ra), CD122 (IL-2Rb), and CD132 (common γc). CD44^−^CD62L^+^Foxp3^−^ cells from 1-wk-old mice had significantly increased expression of CD25 and CD122 compared with adult cells (Fig. S5 D). CD132 was undetectable above the negative control in all cell types. CD25 and CD122 expression was substantially lower than expression of the same proteins on Foxp3^+^ CD4^+^ T cells (Fig. S5 D), with only 20% of CD44^−^CD62L^+^Foxp3^−^ cells expressing CD25 (Fig. S5 E), indicating that this change in receptor expression is unlikely to fully explain the increased sensitivity seen in neonatal CD4^+^ T cells.

These data suggest a predisposition of CD4^+^ T cells for enhanced STAT5 over STAT3 signaling in early life compared with adulthood, with both elevated expression of IL-2 and sensitivity to IL-2 present, potentially limiting T_FH_ cell differentiation in early life.

## Discussion

Antibody-mediated immunity is essential to long-term protection following infection or vaccination but is often defective in early life. Here, we found that T_FH_ are essential for protective immunity after RSV infection but fail to differentiate after early-life infection. The absence of T_FH_ in early life was associated with increased severity of disease on reinfection, and this phenotype was reproduced in T_FH_-deficient adult mice. Strikingly naive CD4 T cells from neonatal mice had a heightened capacity to phosphorylate STAT5 in response to IL-2, and antibody-mediated immunity can be boosted by removing IL-2 signaling during neonatal infection.

In adult mice, RSV infection and other respiratory infections such as IAV (Yoo et al., 2012) robustly promote T_FH_ and corresponding B cell and antibody responses. These T_FH_ responses are essential in protecting adult mice from disease when RSV reinfection occurs. The disease on reinfection seen in *Bcl6*^ΔCD4^ mice, which have a targeted depletion of T_FH_, is very similar to that observed when WT mice receive their first RSV infection as neonates (Culley et al., 2002). Here, we show a critical role for T_FH_ in maintaining immunological balance, and in their presence, viral load, Th1 and Th2 cells, CD8 T cells, eosinophils, and NK cells are limited after RSV reinfection. T_FH_ do not appear to regulate these immune responses during primary infection but likely act predominantly through their role in promoting T-dependent antibody. These antibodies will limit reinfection, in line with the reduced viral loads seen when T_FH_ are present, thus reducing inflammation. In support of this, administration of RSV-specific antibodies before infection has been shown to protect mice from disease (Collarini et al., 2009; Fisher et al., 1999; Habibi et al., 2015; Karron et al., 2013; Radu et al., 2010).

While T_FH_ enable protective immunity during adult RSV infection, their failure to form after early-life infection in mice is associated with an increase in the severity of disease on reinfection. T_FH_ differentiation in neonatal mice has previously been shown to be limited in response to systemic vaccination with ovalbumin/alum or hemagglutinin from IAV (Debock et al., 2013; Mastelic et al., 2012; Mastelic Gavillet et al., 2015). It is important to note, however, that the immunological development of mice does not precisely mirror that observed in humans, with a 1-d-old mouse resembling a premature newborn human infant (Adkins et al., 2004; Simon et al., 2015). At 1 wk of age, mice then more closely resemble human infants at term and rapidly mature, with weaning occurring between 2 and 3 wk of age and reaching adolescence by 6–8 wk of age, as compared with 2 yr and ≥10 yr, respectively, in humans. Thus, the experiments performed here could be considered analogous to comparing infancy to young adolescence in humans. Fitting this comparison, human infants have reduced circulating T_FH_ and plasmablast frequencies compared with older children and poorer response to the RTS,S malaria vaccine (Hill et al., 2020). Lower antibody titers are also seen in response to live attenuated RSV vaccines in infants compared with older age groups (Karron et al., 2013). Infants can also be repeatedly infected with the same serotype of RSV before the age of 3 yr, suggesting infection is insufficient to generate protective titers of antibodies (Openshaw et al., 2017), thus highlighting the significance and importance of defects in T_FH_ differentiation in infancy. Caution should, however, be taken directly translating the findings observed here directly to human RSV infection. A substantially higher dose of RSV is required to infect a mouse than would be necessary to establish an infection in a human (Habibi et al., 2015; Pribul et al., 2008). Further, unlike a proportion of human infants, neonatal mice do not develop severe bronchiolitis upon RSV exposure, and indeed, as also shown here, they display less severe disease, as measured by weight loss, than mice infected with RSV as adults, though this may be a function of how infection interferes with appetite (Groves et al., 2020).

The comparison of neonatal and adult RSV infection in mice does, however, allow a detailed exploration of the mechanisms regulating T_FH_ differentiation during respiratory virus infection at different ages and their implications in a well-characterized and relevant setting. In neonatal mice, defective T_FH_ differentiation after protein immunization can be partially overcome by use of a suitable adjuvant (Mastelic-Gavillet et al., 2019; Mastelic Gavillet et al., 2015; Vono et al., 2018). Here, we show that live RSV infection, despite possessing multiple stimulatory pathogen-associated molecular patterns and damage-associated molecular patterns, does not promote T_FH_ differentiation in neonates. This is despite the presence of a similar frequency of virus-specific CD4^+^ T cells in neonates to that seen after adult infection, suggesting it is not simply due to reduced antigen presentation. Nor does it appear to be the results of enhanced T_FR_ suppressing formation of T_FH_. Likewise, preexposure of neonatal mice to CpG before RSV infection is not sufficient to increase antibody responses (Yamaguchi et al., 2012). The limited generation of T_FH_ in early life also appears to be independent of mouse strain, with both BALB/c and C57BL/6 background failing to generate T_FH_ and antibody responses after neonatal RSV infection. Limited protective immunity after early-life RSV infection is a common feature across many, although interestingly not all, different murine genetic backgrounds (Tregoning et al., 2010), in line with this observation.

The few T_FH_ that are induced in neonatal RSV infection display similar features to those observed after protein vaccination of neonatal mice, with reduced expression of Bcl6, CXCR5, and PD-1. This particular expression pattern has been shown to lead to preferential support of T-independent rather than T-dependent antibody responses in other models (Mastelic-Gavillet et al., 2019). In vaccination, this dysfunction has been associated with excess IL-4 signaling in early life (Debock et al., 2013), and fitting with this, neonatal exposure to RSV is also associated with enhanced IL-4 signaling (Culley et al., 2002). Overexposure of either neonatal or adult mice to IL-4 during primary infection does not, however, suppress the total amount of RSV-specific antibody generated (Harker et al., 2007; Harker et al., 2010), suggesting IL-4 signaling is not the key signal dysregulating T_FH_ after early-life infection. Conversely, overexpression of IFN-γ, which antagonizes IL-4 production, does limit virus-specific antibody, and its neutralization during neonatal RSV infection leads to increased antibody responses (Harker et al., 2007; Harker et al., 2010; Tregoning et al., 2013). Interestingly, here, we find that IFN-γ does not limit T_FH_ differentiation, as has been seen in malaria infection (Ryg-Cornejo et al., 2016), but rather suppresses overall adaptive immune responses.

Initial T_FH_ differentiation from naive CD4 T cells is known to rely on STAT3 signaling; in mice, this is driven through gp130-associated cytokines such as IL-6 (Harker et al., 2013; McIlwain et al., 2015; Ray et al., 2014). STAT5, which is potently activated by IL-2, antagonizes this process preventing T_FH_ differentiation and favoring the differentiation of other CD4^+^ T cell subsets (Ballesteros-Tato et al., 2012; Johnston et al., 2012; Nurieva et al., 2012). While several other studies have shown the capacity of IL-2 to limit T_FH_ differentiation in adult mice, here, we show for the first time that T_FH_ differentiation during early-life infection is found to be exquisitely suppressed by IL-2, with virtually no T_FH_ found in its presence. The removal of IL-2 during neonatal RSV infection leads to increased T_FH_ formation, GC reactions, and virus-specific antibody and is associated with increased protective immunity on reinfection in adulthood.

IL-2 is among the first cytokines produced upon TCR engagement in a naive CD4^+^ T cell. Given the potent ability of IL-2 to suppress T cell–dependent antibody responses, adult mouse models have highlighted that IL-2 signaling is therefore tightly regulated at multiple levels to enable T_FH_ differentiation, including DC-dependent quenching of IL-2 through expression of CD25 (Li et al., 2016) and T cell desensitization (DiToro et al., 2018). We, however, find enhanced *Il2* expression in the LNs of neonatal mice after viral infection compared with adults. This is associated with neonatal Foxp3^−^ CD4^+^ T cells possessing a significantly increased capacity to produce IL-2 both before and after infection. The increased IL-2 in the neonatal LN appears to impede T_FH_ differentiation, and pSTAT5 is increased in activated neonatal CD4^+^ T cells compared with adult CD4^+^ T cells while pSTAT3 is decreased. Suppressing IL-2 leads to increased T_FH_ frequencies at this early time point. We also found an alteration in the ability of neonatal naive CD4^+^ T cells to respond to IL-2 signaling, with STAT5 more readily phosphorylated in response to IL-2 as compared with adult-derived CD4^+^ T cells showing an intrinsic difference between the naive CD4 T cells at an early age compared with adulthood.

The precise mechanism underlying this enhanced IL-2 signaling in neonatal LNs remains unknown. It has been proposed that T reg cells and self-reactive CD4^+^ T cells participate in quorum sensing, coordinated by IL-2, whereby self-reactive CD4^+^ T cells promote T reg cell development and function, suppressing self-reactive T cell activity (Almeida et al., 2012; Amado et al., 2013). IL-2 from non–T reg cells is thought to be essential in the peripheral maintenance of T reg cells (Setoguchi et al., 2005), while IL-2 produced by single-positive CD4^+^ thymocytes is key in regulating T reg cell generation (Hemmers et al., 2019). In mice, the first few weeks of life are associated with rapid thymic output and the establishment of peripheral tolerance, including the progressive generation of Foxp3^+^ CD4 T cells (Fontenot et al., 2005). Fate-mapping of IL-2 also indicates the rapid accumulation IL-2–producing CD4 T cells in the periphery during this time period (Yamamoto et al., 2013), in line with the enhanced IL-2 production observed in the dLNs. It seems likely, at least in part, therefore that the increase in IL-2, and its suppression of T_FH_, seen in early life is partially a result of the requirement for IL-2 signaling in the establishment of peripheral tolerance to self-reactive and microbial antigens in early life. This may explain why, unlike in adulthood (Setoguchi et al., 2005), the failure of IL-2 neutralizing antibody administration in early life to alter T reg cell numbers p.i. It also highlights the potential challenges encountered when seeking to modulate this signaling pathway in early life.

Overall, this study demonstrates that protective antibody-mediated immunity against respiratory viral infection is dependent on T_FH_ and is limited in early life. Enhanced IL-2 signaling in CD4^+^ T cells in neonatal mice prevents the differentiation of T_FH_, and their absence results in significant disease on reinfection. While it remains to be seen if this precise mechanism is in action in human infants infected with RSV, this age group frequently experiences recurrent infection with a variety of pathogens in early life, and immunizations often display reduced efficacy. The recent observation of reduced T_FH_ responses in infants being immunized during the postnatal period further supports a similar process occurring in human babies (Hill et al., 2020). Identifying vaccine strategies that promote STAT3 signals while limiting STAT5 activation and ultimately favor T_FH_ generation could be an important strategy in enhancing infant immunity.

## Materials and methods

### Animals

All animal work was conducted in accordance with the UK Home Office and the local AWERB committee (UK Home Office project license P996A24e1). Specific pathogen–free 6- to 8-wk-old or pregnant female BALB/c and C57BL/6 mice were purchased from Charles River Laboratories. 6- to 10-wk-old B6.129S-*Bcl6^tm1.1Dent^*/J (Bcl6^fl^) mice (Hollister et al., 2013; stock number 023727) were purchased from The Jackson Laboratory and bred with B6.Cg-Tg(Cd4-cre)1Cwi/BfluJ (CD4Cre) mice (Lee et al., 2001; stock number 022071; kindly provided by Professor Matthias Merkenschlager, Imperial College London, London, UK) at Central Biomedical Services (Imperial College London, UK) and Charles River Laboratories (Margate, UK). Genotypes were determined using DNA extracted from ear biopsies processed using Extract-N-Amp Tissue PCR Kit (Sigma-Aldrich), amplified by PCR, and separated by gel electrophoresis according to the Bcl6^fl^ and Generic Cre protocols provided by The Jackson Laboratory; custom PCR primers were purchased from Sigma-Aldrich.

### In vivo viral infections

Plaque-purified RSV A2 was obtained either from ATCC or kindly provided by Dr Cecilia Johansson (Imperial College London, London, UK). RSV A2 was grown in HEp-2 cells and viral titer determined by focus-forming assay (Pribul et al., 2008). WT BALB/c mice were infected i.n. at 1, 2, or 8 wk of age with 2.5 × 10^5^ focus-forming units (ffu) RSV A2 in 20, 40, and 100 µl PBS, respectively, and rechallenged with 10^6^ ffu RSV A2 in 100 µl PBS at 8 wk after primary infection. WT C57BL/6 (*Bcl6^fl/fl^*) or T_FH_-deficient KO (*Cd4*cre *Bcl6^fl/fl^*) mice were infected i.n. at 1 wk or 7–8 wk of age with 2.5 × 10^5^ or 10^6^ ffu RSV A2, respectively. Mice initially infected as adults were rechallenged 4 wk later with 10^6^ ffu RSV A2 i.n.; mice initially infected at 1 wk were rechallenged 8 wk later with 8 × 10^5^ ffu RSV A2.

H1N1 IAV PR8 (strain A/Puerto Rico/8/1934 H1N1) was kindly provided by Professor Wendy Barclay (Imperial College London, London, UK). At 7–8 wk of age, mice were infected i.n. with 40 PFU IAV PR8 and rechallenged with the same dose 4 wk later. All infections were performed under isoflurane-induced anesthesia, and animals were subsequently weighed daily to monitor disease severity. Mice were housed in individually ventilated cages, and all procedures were approved by the UK Home Office and conducted in accordance with the Animals (Scientific Procedures) Act 1986.

### In vivo cytokine depletion

IL-2 was depleted by i.p. administration of 100 µg anti-mouse IL-2 (clones S4B6-1 and JES6-5H4; BioXCell) on days −1, +2, and +5 p.i. Control animals were dosed to the same schedule with 100 µg rat IgG2a (clones LTF-2 and 2A3; BioXCell). IFN-γ was depleted by i.p. administration of 100 µg anti-mouse IFN-γ (clone XMG1.2; BioLegend) on days −1, +2, and +5 p.i., while control animals received 100 µg rat IgG1 (clone HRPN; BioXCell).

### Cell recovery

Mice were exsanguinated via the jugular vein. Blood was collected using heparinized capillary tubes (Hirschmann) and then centrifuged to isolate plasma. Bronchoalveolar lavage was performed by cannulating the trachea and washing the lungs with sterile PBS (1 ml flushed into the airways three times for adult mice and 0.5 ml flushed three times for mice under 5 wk of age). After centrifugation, supernatant was taken for mediator analysis, and cells were resuspended and used for flow cytometry. The right lung lobe was snap frozen and then disrupted in RPMI 1640, 100 U/ml penicillin/streptomycin (Life Technologies) using a handheld homogenizer (Stuart SHM1). Whole homogenate was used to determine viral load and supernatant was used for mediator measurements. All other lung lobes, mediastinal LNs (dLN), and spleen were processed for flow cytometry. Lung tissue was weighed, finely chopped, and incubated for 45 min at 37°C in complete RPMI (RPMI 1640, 10% FCS, 2 mM L-glutamine, and 100 U/ml penicillin/streptomycin; Life Technologies) supplemented with 0.15 mg/ml collagenase D (Roche Diagnostics) and 25 µg/ml DNase I (Roche Diagnostics). Lung, LN, and spleen were passed through 100-µm cell strainers (Greiner Bio-One) to create single-cell suspensions in complete RPMI. Lung and splenic cell suspensions were centrifuged, supernatant removed, and the cells treated with ACK buffer (150 mM ammonium chloride, 10 mM potassium bicarbonate, and 0.1 mM EDTA) for 3 min at room temperature and then washed and resuspended in complete RPMI. Total live cell counts were obtained using trypan blue exclusion staining and FastRead 102 counting slides (Kova International).

### Ex vivo cell stimulations

For detection of T cell cytokine production, single-cell suspensions were stimulated by 5-h incubation with 10 ng/ml PMA (Sigma-Aldrich), 0.5 µg/ml ionomycin (VWR), 10 µg/ml Brefeldin A (Sigma-Aldrich), and 50 U/ml recombinant mouse IL-2 (R&D Systems) in complete RPMI at 37°C. Virus-specific T cell cytokine production was induced by 5-h incubation with RSV peptides, including 3 µg/ml CD8 stimulating M2_82–90_ (SYIGSINNI) and F_85–93_ (KYKNAVTEL) and 5 µg/ ml CD4 stimulating G_181–197_ (TCWAICKRIPNKKPGKK), F_51–66_ (GWYTSVITIELSNIKE), and P_39–55_ (SIISVNSIDIEVTKESP) for BALB/c samples and 3 µg/ ml CD8 stimulating M2_187–195_ (NAITNAKII) with 10 µg/ml CD4 stimulating M_209–223_ (NKGAFKYIKPQSQFI) and M2-1_26–39_ (NYFEWPPHALLVRQ) for BL/6 samples.

For detection of STAT phosphorylation, ex vivo tissues were harvested directly into warm 1% paraformaldehyde (PFA) in PBS and incubated for 10 min at 37°C. A single-cell suspension was then generated for subsequent staining. For detection of STAT phosphorylation following in vitro cytokine stimulation, single-cell suspensions were rested in RPMI 1640 and 100 U/ml penicillin/streptomycin (Life Technologies) for 2 h and then stimulated with 150 U/ml rIL-2 or 50 ng/ml rIL-6 (both from BioLegend) for 15 min. Cells were then fixed with 1% PFA (Sigma-Aldrich) in PBS for 15 min. All steps were performed at 37°C. Cells were subsequently frozen in True-Phos Perm Buffer (BioLegend) at −80°C until processed as described for flow cytometry.

### Flow cytometry

Flow cytometry was performed as described previously (Pyle et al., 2017). All antibodies are from BioLegend unless otherwise stated. Single-cell suspensions were first stained with UV fixable viability dye (BioLegend) in PBS for 20 min at 4°C. The following anti-mouse antibodies were diluted in PBS containing 2% fetal bovine serum (Life Technologies) and 2 mM EDTA and used for surface staining, in combination with a 1 in 100 dilution of TruStain FcX antibody (BioLegend): anti-CD4 (RM4-5), anti-CD3e (145-2C11; eBioscience, Life Technologies); anti-CD8 (53–6.7), anti-B220 (RA3-6B2), anti-CD19 (6D5), anti-CD11c (N418; eBioscience); anti-CD11b (M1/70), anti-Ly6C (HK1.4), anti-Ly6G (1A8), anti-CD127 (A7R34), anti-CD45 (30-F11), anti-CD90.2 (53–2.1), anti-Nkp46 (29A1.4), anti-CD64 (X54-5/7.1), anti-Ia/Ie (M5/114.15.2), anti-CD11a (M17/4), anti-CD49d (R1-2; eBioscience); anti-CD44 (IM7), anti-SiglecF (BD), anti-GITR (DTA-1; BD), anti-CD68 (FA-11), anti-CD62L (MEL-14), anti-PSGL1 (2PH1), anti-PD-1 (29F.1A12), anti-CXCR5 (L138D7), anti-ICOS (C398.4A), anti-CD38 (90; eBioscience); anti-GL7 (GL7), anti-Fas (15A7; eBioscience), anti-CD138 (281–2), anti-IgG1 (RMG1-1), anti-IgG2a (RMG2a-62), anti-IgD (11-26c.2a), anti-IgM (RMM-1), anti-KLRG1 (MAFA), anti-CD25 (PC61), CD80 (16-10A1), K^b^ M2_82–90_ (for BALB/c mice), or D^b^ M_187–195_ and I-Ab M2_27–37_ (for C57BL/6 mice) tetramers. Tetramers were kindly provided by the NIH Tetramer Core Facility, either prefolded or as monomers, which were folded in-house with SA-PE (Invitrogen). For intracellular cytokine staining, cells were fixed with 1% PFA (Sigma-Aldrich) in PBS for 10 min at room temperature and stained with the following antibodies in permeabilization buffer (eBioscience): anti-mouse IL-4 (11B11), anti-IL-17A (TC11), anti-IFN-γ (XMG1.2), anti-IL-2 (JES6-5H4), and anti-IL-13 (eBio13A; eBioscience). For intranuclear staining, cells were permeabilized and fixed with Transcription Factor Fixation/Permeabilization Kit (eBioscience) and stained with the following antibodies in permeabilization buffer (eBioscience): anti-mouse-Foxp3 (FJK-16s; eBioscience) and anti-Bcl6 (K112-91; BD). For pSTAT detection, cells were fixed with 1% PFA in PBS for 10 min at room temperature, permeabilized with True-Phos Perm Buffer (BioLegend) at −20°C, incubated with a 1:100 dilution of TruStain FcX antibody for 1 h, and stained with mouse anti-Stat5 (pY694, BD) and anti-Stat3 (pS727; BD) alongside surface-stain antibodies detailed above in permeabilization buffer (eBioscience). Samples were acquired using a five-laser LSRFortessa (BD) and analyzed using FlowJo (BD).

### Immunofluorescence microscopy

Preparation and imaging of dLN and spleen was performed as previously described (Wong et al., 2019). Briefly, tissues were harvested directly into 4% PFA and 5% sucrose in PBS and fixed for 4–6 h and transferred to 10% in PBS for 1 h and then 30% sucrose overnight. All steps were performed at 4°C. Tissues were then frozen in optimal cutting temperature compound, and 8-µm cryostat sections were prepared, fixed with 4% PFA, and blocked with blocking solution (PBS containing 3% normal goat serum, 3% normal donkey serum, 1% BSA, 0.05% Tween 20, and 0.1% Triton X-100). Sections were then stained with rat anti-mouse IgD-Alexa Fluor 647 (11-26c2a), rat anti-mouse/human GL7-Alexa488 (GL7), and anti-mouse CD4-biotin (RM4-5) diluted in blocking solution for 2 h at room temperature, washed, incubated with streptavidin-BV421 diluted in blocking solution for 2 h, washed, refixed with 4% PFA, and mounted. Sections were imaged using a Leica SP5 MP/FLIM inverted microscope and images analyzed in using FIJI software.

### Quantitative RT-PCR

RNA was extracted from lung, dLN, and spleen homogenate using RNeasy Mini Kit columns (Qiagen) according to the manufacturer’s instructions and converted to cDNA using High-Capacity cDNA Reverse Transcription Kit (ThermoFisher) or GoScript Reverse transcription Kit (Promega). TaqMan assays (ThermoFisher) were used to assess the expression of *IL2* and *IL6* relative to *Gapdh*. RSV L gene was quantified relative to 18s housekeeping gene using a 900 nM forward primer (5′-GAA​CTC​AGT​GTA​GGT​AGA​ATG​TTT​GCA-3′), 300 nM reverse primer (5′-TTC​AGC​TAT​CAT​TTT​CTC​TGC​CAA​T-3′), and 175 nM probe (5′-6-carboxyfluorescein-TTTGAACCTGTCTGAACATTCCCGGTT-6-carboxytetramethylrhodamine-3′) as described in Harker et al. (2014). Quantitative RT-PCR was performed using a Viia7 RT-PCR System (ThermoFisher).

### Antibody and cytokine measurements

IgE, IL-4, IL-13, and IFN-γ were measured using sandwich ELISA kits (Invitrogen) according to manufacturer’s instructions. For quantification of virus-specific Ig, IgG1, IgG2a/c, IgA, and IgM, ELISA plates were coated with RSV antigen grown in the laboratory, and bound antibody was detected using the SBA Clonotyping System-HRP (Southern Biotech).

### Statistics

GraphPad 6.0 (GraphPad) was used for all statistics. For weight loss, area under the curve was calculated and significance tested by a Mann–Whitney *U* test (data with two groups) or one-way ANOVA (data with more than two groups). For experiments with only two groups, a nonparametric Mann–Whitney *U* test was used. For groups with three or more groups, a nonparametric Kruskal–Wallis H test with a Dunn’s test was used. In all cases *, P < 0.05; **, P < 0.01; and ***, P < 0.001.

### Online supplemental material

Fig. S1 demonstrates that adult GC B cell and T_FH_ responses peak at day 14 after RSV infection, with local and systemic antibody responses increasing between 1 and 3 wk p.i. In contrast, adaptive immune responses and GC formation is significantly reduced in mice infected as neonates. Fig. S2 shows that Bcl6 deficiency in T cells restricts antibody responses to IAV after primary infection but does not result in enhanced disease upon reinfection. Fig. S3 illustrates that the immune response to primary RSV infection in neonatal mice is not dependent on Bcl6 expression in T cells, using WT (*Bcl6^fl/fl^*) and T_FH_-deficient KO (*Cd4*cre *Bcl6^fl/fl^*) mice. Fig. S4 details that neutralization of IL-2 during neonatal RSV infection limits lung and airway inflammation upon rechallenge with RSV in a Bcl6-dependent manner and does not affect numbers of T reg cells or T_FR_. Fig. S5 demonstrates that CD8^+^ T cells and Foxp3^−^CD4^+^ T cells are the main source of IL-2 in neonatal secondary lymphoid tissue following RSV infection and that IL-2 receptor subunit expression is increased on neonatal naive Foxp3^−^CD4^+^ T cells, compared with adults.

## References

[bib1] Adkins, B., C. Leclerc, and S. Marshall-Clarke. 2004. Neonatal adaptive immunity comes of age. Nat. Rev. Immunol. 4:553–564. 10.1038/nri1394 15229474

[bib2] Almeida, A.R., I.F. Amado, J. Reynolds, J. Berges, G. Lythe, C. Molina-París, and A.A. Freitas. 2012. Quorum-Sensing in CD4(+) T Cell Homeostasis: A Hypothesis and a Model. Front. Immunol. 3:125. 10.3389/fimmu.2012.00125 22654881 PMC3360200

[bib3] Amado, I.F., J. Berges, R.J. Luther, M.P. Mailhé, S. Garcia, A. Bandeira, C. Weaver, A. Liston, and A.A. Freitas. 2013. IL-2 coordinates IL-2-producing and regulatory T cell interplay. J. Exp. Med. 210:2707–2720. 10.1084/jem.20122759 24249704 PMC3832933

[bib4] Ballesteros-Tato, A., B. León, B.A. Graf, A. Moquin, P.S. Adams, F.E. Lund, and T.D. Randall. 2012. Interleukin-2 inhibits germinal center formation by limiting T follicular helper cell differentiation. Immunity. 36:847–856. 10.1016/j.immuni.2012.02.012 22464171 PMC3361521

[bib5] Choi, Y.S., R. Kageyama, D. Eto, T.C. Escobar, R.J. Johnston, L. Monticelli, C. Lao, and S. Crotty. 2011. ICOS receptor instructs T follicular helper cell versus effector cell differentiation via induction of the transcriptional repressor Bcl6. Immunity. 34:932–946. 10.1016/j.immuni.2011.03.023 21636296 PMC3124577

[bib6] Choi, Y.S., D. Eto, J.A. Yang, C. Lao, and S. Crotty. 2013. Cutting edge: STAT1 is required for IL-6-mediated Bcl6 induction for early follicular helper cell differentiation. J. Immunol. 190:3049–3053. 10.4049/jimmunol.1203032 23447690 PMC3626564

[bib7] Collarini, E.J., F.E. Lee, O. Foord, M. Park, G. Sperinde, H. Wu, W.D. Harriman, S.F. Carroll, S.L. Ellsworth, L.J. Anderson, . 2009. Potent high-affinity antibodies for treatment and prophylaxis of respiratory syncytial virus derived from B cells of infected patients. J. Immunol. 183:6338–6345. 10.4049/jimmunol.0901373 19841167

[bib8] Culley, F.J., J. Pollott, and P.J. Openshaw. 2002. Age at first viral infection determines the pattern of T cell-mediated disease during reinfection in adulthood. J. Exp. Med. 196:1381–1386. 10.1084/jem.20020943 12438429 PMC2193991

[bib9] Debock, I., K. Jaworski, H. Chadlaoui, S. Delbauve, N. Passon, L. Twyffels, O. Leo, and V. Flamand. 2013. Neonatal follicular Th cell responses are impaired and modulated by IL-4. J. Immunol. 191:1231–1239. 10.4049/jimmunol.1203288 23804713

[bib10] DiToro, D., C.J. Winstead, D. Pham, S. Witte, R. Andargachew, J.R. Singer, C.G. Wilson, C.L. Zindl, R.J. Luther, D.J. Silberger, . 2018. Differential IL-2 expression defines developmental fates of follicular versus nonfollicular helper T cells. Science. 361:eaao2933. 10.1126/science.aao2933 30213884 PMC6501592

[bib11] Dolence, J.J., T. Kobayashi, K. Iijima, J. Krempski, L.Y. Drake, A.L. Dent, and H. Kita. 2018. Airway exposure initiates peanut allergy by involving the IL-1 pathway and T follicular helper cells in mice. J. Allergy Clin. Immunol. 142:1144–1158.e8. 10.1016/j.jaci.2017.11.020 29247716 PMC6002896

[bib12] Fisher, R.G., J.E. Johnson, S.B. Dillon, R.A. Parker, and B.S. Graham. 1999. Prophylaxis with respiratory syncytial virus F-specific humanized monoclonal antibody delays and moderately suppresses the native antibody response but does not impair immunity to late rechallenge. J. Infect. Dis. 180:708–713. 10.1086/314965 10438358

[bib13] Fontenot, J.D., J.L. Dooley, A.G. Farr, and A.Y. Rudensky. 2005. Developmental regulation of Foxp3 expression during ontogeny. J. Exp. Med. 202:901–906. 10.1084/jem.20050784 16203863 PMC2213175

[bib14] Greczmiel, U., N.J. Kräutler, A. Pedrioli, I. Bartsch, P. Agnellini, G. Bedenikovic, J. Harker, K. Richter, and A. Oxenius. 2017. Sustained T follicular helper cell response is essential for control of chronic viral infection. Sci. Immunol. 2:eaam8686. 10.1126/sciimmunol.aam8686 29196449

[bib15] Grimbacher, B., A. Hutloff, M. Schlesier, E. Glocker, K. Warnatz, R. Dräger, H. Eibel, B. Fischer, A.A. Schäffer, H.W. Mages, . 2003. Homozygous loss of ICOS is associated with adult-onset common variable immunodeficiency. Nat. Immunol. 4:261–268. 10.1038/ni902 12577056

[bib16] Groves, H.T., S.L. Higham, M.F. Moffatt, M.J. Cox, and J.S. Tregoning. 2020. Respiratory Viral Infection Alters the Gut Microbiota by Inducing Inappetence. MBio. 11:11. 10.1128/mBio.03236-19 PMC7029140 32071269

[bib17] Habibi, M.S., A. Jozwik, S. Makris, J. Dunning, A. Paras, J.P. DeVincenzo, C.A. de Haan, J. Wrammert, P.J. Openshaw, and C. Chiu. Mechanisms of Severe Acute Influenza Consortium Investigators. 2015. Impaired Antibody-mediated Protection and Defective IgA B-Cell Memory in Experimental Infection of Adults with Respiratory Syncytial Virus. Am. J. Respir. Crit. Care Med. 191:1040–1049. 10.1164/rccm.201412-2256OC 25730467 PMC4435460

[bib18] Harker, J., A. Bukreyev, P.L. Collins, B. Wang, P.J. Openshaw, and J.S. Tregoning. 2007. Virally delivered cytokines alter the immune response to future lung infections. J. Virol. 81:13105–13111. 10.1128/JVI.01544-07 17855518 PMC2169117

[bib19] Harker, J.A., D.C. Lee, Y. Yamaguchi, B. Wang, A. Bukreyev, P.L. Collins, J.S. Tregoning, and P.J. Openshaw. 2010. Delivery of cytokines by recombinant virus in early life alters the immune response to adult lung infection. J. Virol. 84:5294–5302. 10.1128/JVI.02503-09 20200251 PMC2863826

[bib20] Harker, J.A., G.M. Lewis, L. Mack, and E.I. Zuniga. 2011. Late interleukin-6 escalates T follicular helper cell responses and controls a chronic viral infection. Science. 334:825–829. 10.1126/science.1208421 21960530 PMC3388900

[bib21] Harker, J.A., A. Dolgoter, and E.I. Zuniga. 2013. Cell-intrinsic IL-27 and gp130 cytokine receptor signaling regulates virus-specific CD4^+^ T cell responses and viral control during chronic infection. Immunity. 39:548–559. 10.1016/j.immuni.2013.08.010 23993651 PMC4701058

[bib22] Harker, J.A., Y. Yamaguchi, F.J. Culley, J.S. Tregoning, and P.J. Openshaw. 2014. Delayed sequelae of neonatal respiratory syncytial virus infection are dependent on cells of the innate immune system. J. Virol. 88:604–611. 10.1128/JVI.02620-13 24173217 PMC3911760

[bib23] Harker, J.A., K.A. Wong, A. Dolgoter, and E.I. Zuniga. 2015. Cell-Intrinsic gp130 Signaling on CD4+ T Cells Shapes Long-Lasting Antiviral Immunity. J. Immunol. 195:1071–1081. 10.4049/jimmunol.1402402 26085685 PMC4506854

[bib24] He, J., L.M. Tsai, Y.A. Leong, X. Hu, C.S. Ma, N. Chevalier, X. Sun, K. Vandenberg, S. Rockman, Y. Ding, . 2013. Circulating precursor CCR7(lo)PD-1(hi) CXCR5^+^ CD4^+^ T cells indicate Tfh cell activity and promote antibody responses upon antigen reexposure. Immunity. 39:770–781. 10.1016/j.immuni.2013.09.007 24138884

[bib25] Hemmers, S., M. Schizas, E. Azizi, S. Dikiy, Y. Zhong, Y. Feng, G. Altan-Bonnet, and A.Y. Rudensky. 2019. IL-2 production by self-reactive CD4 thymocytes scales regulatory T cell generation in the thymus. J. Exp. Med. 216:2466–2478. 10.1084/jem.20190993 31434685 PMC6829602

[bib26] Hill, D.L., E.J. Carr, T. Rutishauser, G. Moncunill, J.J. Campo, S. Innocentin, M. Mpina, A. Nhabomba, A. Tumbo, C. Jairoce, . 2020. Immune system development varies according to age, location, and anemia in African children. Sci. Transl. Med. 12: eaaw9522. 10.1126/scitranslmed.aaw9522 32024802 PMC7738197

[bib27] Hollister, K., S. Kusam, H. Wu, N. Clegg, A. Mondal, D.V. Sawant, and A.L. Dent. 2013. Insights into the role of Bcl6 in follicular Th cells using a new conditional mutant mouse model. J. Immunol. 191:3705–3711. 10.4049/jimmunol.1300378 23980208 PMC3783642

[bib28] Johnston, R.J., A.C. Poholek, D. DiToro, I. Yusuf, D. Eto, B. Barnett, A.L. Dent, J. Craft, and S. Crotty. 2009. Bcl6 and Blimp-1 are reciprocal and antagonistic regulators of T follicular helper cell differentiation. Science. 325:1006–1010. 10.1126/science.1175870 19608860 PMC2766560

[bib29] Johnston, R.J., Y.S. Choi, J.A. Diamond, J.A. Yang, and S. Crotty. 2012. STAT5 is a potent negative regulator of TFH cell differentiation. J. Exp. Med. 209:243–250. 10.1084/jem.20111174 22271576 PMC3281266

[bib30] Karron, R.A., U.J. Buchholz, and P.L. Collins. 2013. Live-attenuated respiratory syncytial virus vaccines. Curr. Top. Microbiol. Immunol. 372:259–284. 24362694 10.1007/978-3-642-38919-1_13 PMC4794267

[bib31] Lee, P.P., D.R. Fitzpatrick, C. Beard, H.K. Jessup, S. Lehar, K.W. Makar, M. Pérez-Melgosa, M.T. Sweetser, M.S. Schlissel, S. Nguyen, . 2001. A critical role for Dnmt1 and DNA methylation in T cell development, function, and survival. Immunity. 15:763–774. 10.1016/S1074-7613(01)00227-8 11728338

[bib32] Li, J., E. Lu, T. Yi, and J.G. Cyster. 2016. EBI2 augments Tfh cell fate by promoting interaction with IL-2-quenching dendritic cells. Nature. 533:110–114. 10.1038/nature17947 27147029 PMC4883664

[bib33] Linterman, M.A., A.E. Denton, D.P. Divekar, I. Zvetkova, L. Kane, C. Ferreira, M. Veldhoen, S. Clare, G. Dougan, M. Espéli, and K.G. Smith. 2014. CD28 expression is required after T cell priming for helper T cell responses and protective immunity to infection. eLife. 3:e03180. 10.7554/eLife.03180 PMC4241536 25347065

[bib34] Locci, M., C. Havenar-Daughton, E. Landais, J. Wu, M.A. Kroenke, C.L. Arlehamn, L.F. Su, R. Cubas, M.M. Davis, A. Sette, . International AIDS Vaccine Initiative Protocol C Principal Investigators. 2013. Human circulating PD-1+CXCR3-CXCR5+ memory Tfh cells are highly functional and correlate with broadly neutralizing HIV antibody responses. Immunity. 39:758–769. 10.1016/j.immuni.2013.08.031 24035365 PMC3996844

[bib35] Ma, C.S., D.T. Avery, A. Chan, M. Batten, J. Bustamante, S. Boisson-Dupuis, P.D. Arkwright, A.Y. Kreins, D. Averbuch, D. Engelhard, . 2012. Functional STAT3 deficiency compromises the generation of human T follicular helper cells. Blood. 119:3997–4008. 10.1182/blood-2011-11-392985 22403255 PMC3355712

[bib36] Malek, T.R., and I. Castro. 2010. Interleukin-2 receptor signaling: at the interface between tolerance and immunity. Immunity. 33:153–165. 10.1016/j.immuni.2010.08.004 20732639 PMC2946796

[bib37] Mastelic, B., A.T. Kamath, P. Fontannaz, C. Tougne, A.F. Rochat, E. Belnoue, C. Combescure, F. Auderset, P.H. Lambert, F. Tacchini-Cottier, and C.A. Siegrist. 2012. Environmental and T cell-intrinsic factors limit the expansion of neonatal follicular T helper cells but may be circumvented by specific adjuvants. J. Immunol. 189:5764–5772. 10.4049/jimmunol.1201143 23162125

[bib38] Mastelic Gavillet, B., C.S. Eberhardt, F. Auderset, F. Castellino, A. Seubert, J.S. Tregoning, P.H. Lambert, E. de Gregorio, G. Del Giudice, and C.A. Siegrist. 2015. MF59 Mediates Its B Cell Adjuvanticity by Promoting T Follicular Helper Cells and Thus Germinal Center Responses in Adult and Early Life. J. Immunol. 194:4836–4845. 10.4049/jimmunol.1402071 25870238

[bib39] Mastelic-Gavillet, B., M. Vono, P. Gonzalez-Dias, F.M. Ferreira, L. Cardozo, P.H. Lambert, H.I. Nakaya, and C.A. Siegrist. 2019. Neonatal T Follicular Helper Cells Are Lodged in a Pre-T Follicular Helper Stage Favoring Innate Over Adaptive Germinal Center Responses. Front. Immunol. 10:1845. 10.3389/fimmu.2019.01845 31456798 PMC6700230

[bib40] McIlwain, D.R., M. Grusdat, V.I. Pozdeev, H.C. Xu, P. Shinde, C. Reardon, Z. Hao, M. Beyer, A. Bergthaler, D. Häussinger, . 2015. T-cell STAT3 is required for the maintenance of humoral immunity to LCMV. Eur. J. Immunol. 45:418–427. 10.1002/eji.201445060 25393615 PMC4383653

[bib41] Miyauchi, K., A. Sugimoto-Ishige, Y. Harada, Y. Adachi, Y. Usami, T. Kaji, K. Inoue, H. Hasegawa, T. Watanabe, A. Hijikata, . 2016. Protective neutralizing influenza antibody response in the absence of T follicular helper cells. Nat. Immunol. 17:1447–1458. 10.1038/ni.3563 27798619

[bib42] Nurieva, R.I., Y. Chung, G.J. Martinez, X.O. Yang, S. Tanaka, T.D. Matskevitch, Y.H. Wang, and C. Dong. 2009. Bcl6 mediates the development of T follicular helper cells. Science. 325:1001–1005. 10.1126/science.1176676 19628815 PMC2857334

[bib43] Nurieva, R.I., A. Podd, Y. Chen, A.M. Alekseev, M. Yu, X. Qi, H. Huang, R. Wen, J. Wang, H.S. Li, . 2012. STAT5 protein negatively regulates T follicular helper (Tfh) cell generation and function. J. Biol. Chem. 287:11234–11239. 10.1074/jbc.M111.324046 22318729 PMC3322890

[bib44] Openshaw, P.J.M., C. Chiu, F.J. Culley, and C. Johansson. 2017. Protective and Harmful Immunity to RSV Infection. Annu. Rev. Immunol. 35:501–532. 10.1146/annurev-immunol-051116-052206 28226227

[bib45] Papillion, A., M.D. Powell, D.A. Chisolm, H. Bachus, M.J. Fuller, A.S. Weinmann, A. Villarino, J.J. O’Shea, B. León, K.J. Oestreich, and A. Ballesteros-Tato. 2019. Inhibition of IL-2 responsiveness by IL-6 is required for the generation of GC-T_FH_ cells. Sci. Immunol. 4:eaaw7636. 10.1126/sciimmunol.aaw7636 31519812 PMC6820141

[bib46] Poholek, A.C., K. Hansen, S.G. Hernandez, D. Eto, A. Chandele, J.S. Weinstein, X. Dong, J.M. Odegard, S.M. Kaech, A.L. Dent, . 2010. In vivo regulation of Bcl6 and T follicular helper cell development. J. Immunol. 185:313–326. 10.4049/jimmunol.0904023 20519643 PMC2891136

[bib47] Pribul, P.K., J. Harker, B. Wang, H. Wang, J.S. Tregoning, J. Schwarze, and P.J. Openshaw. 2008. Alveolar macrophages are a major determinant of early responses to viral lung infection but do not influence subsequent disease development. J. Virol. 82:4441–4448. 10.1128/JVI.02541-07 18287232 PMC2293049

[bib48] Pyle, C.J., F.I. Uwadiae, D.P. Swieboda, and J.A. Harker. 2017. Early IL-6 signalling promotes IL-27 dependent maturation of regulatory T cells in the lungs and resolution of viral immunopathology. PLoS Pathog. 13:e1006640. 10.1371/journal.ppat.1006640 28953978 PMC5633202

[bib49] Radu, G.U., H. Caidi, C. Miao, R.A. Tripp, L.J. Anderson, and L.M. Haynes. 2010. Prophylactic treatment with a G glycoprotein monoclonal antibody reduces pulmonary inflammation in respiratory syncytial virus (RSV)-challenged naive and formalin-inactivated RSV-immunized BALB/c mice. J. Virol. 84:9632–9636. 10.1128/JVI.00451-10 20592094 PMC2937657

[bib50] Ray, J.P., H.D. Marshall, B.J. Laidlaw, M.M. Staron, S.M. Kaech, and J. Craft. 2014. Transcription factor STAT3 and type I interferons are corepressive insulators for differentiation of follicular helper and T helper 1 cells. Immunity. 40:367–377. 10.1016/j.immuni.2014.02.005 24631156 PMC3992517

[bib51] Ruckwardt, T.J., A.M. Malloy, E. Gostick, D.A. Price, P. Dash, J.L. McClaren, P.G. Thomas, and B.S. Graham. 2011. Neonatal CD8 T-cell hierarchy is distinct from adults and is influenced by intrinsic T cell properties in respiratory syncytial virus infected mice. PLoS Pathog. 7:e1002377. 10.1371/journal.ppat.1002377 22144888 PMC3228797

[bib52] Ryg-Cornejo, V., L.J. Ioannidis, A. Ly, C.Y. Chiu, J. Tellier, D.L. Hill, S.P. Preston, M. Pellegrini, D. Yu, S.L. Nutt, . 2016. Severe Malaria Infections Impair Germinal Center Responses by Inhibiting T Follicular Helper Cell Differentiation. Cell Rep. 14:68–81. 10.1016/j.celrep.2015.12.006 26725120

[bib53] Setoguchi, R., S. Hori, T. Takahashi, and S. Sakaguchi. 2005. Homeostatic maintenance of natural Foxp3(+) CD25(+) CD4(+) regulatory T cells by interleukin (IL)-2 and induction of autoimmune disease by IL-2 neutralization. J. Exp. Med. 201:723–735. 10.1084/jem.20041982 15753206 PMC2212841

[bib54] Simon, A.K., G.A. Hollander, and A. McMichael. 2015. Evolution of the immune system in humans from infancy to old age. Proc. Biol. Sci. 282:20143085. 10.1098/rspb.2014.3085 26702035 PMC4707740

[bib55] Tregoning, J.S., Y. Yamaguchi, J. Harker, B. Wang, and P.J. Openshaw. 2008. The role of T cells in the enhancement of respiratory syncytial virus infection severity during adult reinfection of neonatally sensitized mice. J. Virol. 82:4115–4124. 10.1128/JVI.02313-07 18272579 PMC2293007

[bib56] Tregoning, J.S., Y. Yamaguchi, B. Wang, D. Mihm, J.A. Harker, E.S. Bushell, M. Zheng, G. Liao, G. Peltz, and P.J. Openshaw. 2010. Genetic susceptibility to the delayed sequelae of neonatal respiratory syncytial virus infection is MHC dependent. J. Immunol. 185:5384–5391. 10.4049/jimmunol.1001594 20921522

[bib57] Tregoning, J.S., B.L. Wang, J.U. McDonald, Y. Yamaguchi, J.A. Harker, M. Goritzka, C. Johansson, A. Bukreyev, P.L. Collins, and P.J. Openshaw. 2013. Neonatal antibody responses are attenuated by interferon-γ produced by NK and T cells during RSV infection. Proc. Natl. Acad. Sci. USA. 110:5576–5581. 10.1073/pnas.1214247110 23509276 PMC3619373

[bib58] Uwadiae, F.I., C.J. Pyle, S.A. Walker, C.M. Lloyd, and J.A. Harker. 2019. Targeting the ICOS/ICOS-L pathway in a mouse model of established allergic asthma disrupts T follicular helper cell responses and ameliorates disease. Allergy. 74:650–662. 10.1111/all.13602 30220084 PMC6492018

[bib59] Vinuesa, C.G., M.A. Linterman, D. Yu, and I.C. MacLennan. 2016. Follicular Helper T Cells. Annu. Rev. Immunol. 34:335–368. 10.1146/annurev-immunol-041015-055605 26907215

[bib60] Vono, M., C.S. Eberhardt, E. Mohr, F. Auderset, D. Christensen, M. Schmolke, R. Coler, A. Meinke, P. Andersen, P.H. Lambert, . 2018. Overcoming the Neonatal Limitations of Inducing Germinal Centers through Liposome-Based Adjuvants Including C-Type Lectin Agonists Trehalose Dibehenate or Curdlan. Front. Immunol. 9:381. 10.3389/fimmu.2018.00381 29541075 PMC5835515

[bib61] Weber, J.P., F. Fuhrmann, R.K. Feist, A. Lahmann, M.S. Al Baz, L.J. Gentz, D. Vu Van, H.W. Mages, C. Haftmann, R. Riedel, . 2015. ICOS maintains the T follicular helper cell phenotype by down-regulating Krüppel-like factor 2. J. Exp. Med. 212:217–233. 10.1084/jem.20141432 25646266 PMC4322049

[bib62] Wong, K.A., J.A. Harker, A. Dolgoter, N. Marooki, and E.I. Zuniga. 2019. T Cell-Intrinsic IL-6R Signaling Is Required for Optimal ICOS Expression and Viral Control during Chronic Infection. J. Immunol. 203:1509–1520. 10.4049/jimmunol.1801567 31413107 PMC8131195

[bib63] Wu, Y., M. Borde, V. Heissmeyer, M. Feuerer, A.D. Lapan, J.C. Stroud, D.L. Bates, L. Guo, A. Han, S.F. Ziegler, . 2006. FOXP3 controls regulatory T cell function through cooperation with NFAT. Cell. 126:375–387. 10.1016/j.cell.2006.05.042 16873067

[bib64] Wu, T., Y. Hu, Y.T. Lee, K.R. Bouchard, A. Benechet, K. Khanna, and L.S. Cauley. 2014. Lung-resident memory CD8 T cells (TRM) are indispensable for optimal cross-protection against pulmonary virus infection. J. Leukoc. Biol. 95:215–224. 10.1189/jlb.0313180 24006506 PMC3896663

[bib65] Wu, H., L.L. Xu, P. Teuscher, H. Liu, M.H. Kaplan, and A.L. Dent. 2015. An Inhibitory Role for the Transcription Factor Stat3 in Controlling IL-4 and Bcl6 Expression in Follicular Helper T Cells. J. Immunol. 195:2080–2089. 10.4049/jimmunol.1500335 26188063 PMC4546859

[bib66] Yamaguchi, Y., J.A. Harker, B. Wang, P.J. Openshaw, J.S. Tregoning, and F.J. Culley. 2012. Preexposure to CpG protects against the delayed effects of neonatal respiratory syncytial virus infection. J. Virol. 86:10456–10461. 10.1128/JVI.01082-12 22811525 PMC3457284

[bib67] Yamamoto, M., Y. Seki, K. Iwai, I. Ko, A. Martin, N. Tsuji, S. Miyagawa, R.B. Love, and M. Iwashima. 2013. Ontogeny and localization of the cells produce IL-2 in healthy animals. Cytokine. 61:831–841. 10.1016/j.cyto.2012.11.026 23332616 PMC3595346

[bib68] Yoo, J.K., E.N. Fish, and T.J. Braciale. 2012. LAPCs promote follicular helper T cell differentiation of Ag-primed CD4+ T cells during respiratory virus infection. J. Exp. Med. 209:1853–1867. 10.1084/jem.20112256 22987801 PMC3457726

[bib69] Yu, D., S. Rao, L.M. Tsai, S.K. Lee, Y. He, E.L. Sutcliffe, M. Srivastava, M. Linterman, L. Zheng, N. Simpson, . 2009. The transcriptional repressor Bcl-6 directs T follicular helper cell lineage commitment. Immunity. 31:457–468. 10.1016/j.immuni.2009.07.002 19631565

